# Incorporation of multiple supramolecular binding sites into a robust MOF for benchmark one-step ethylene purification

**DOI:** 10.1038/s41467-023-41692-x

**Published:** 2023-10-02

**Authors:** Enyu Wu, Xiao-Wen Gu, Di Liu, Xu Zhang, Hui Wu, Wei Zhou, Guodong Qian, Bin Li

**Affiliations:** 1 State Key Laboratory of Silicon and Advanced Semiconductor Materials, School of Materials Science and Engineering, Zhejiang University, Hangzhou, 310027 China; 2 School of Chemistry and Chemical Engineering, Huaiyin Normal University, Huaian, 223300 China; 3 NIST Center for Neutron Research, National Institute of Standards and Technology, Gaithersburg, MD 20899-6102 USA

**Keywords:** Metal-organic frameworks, Porous materials, Metal-organic frameworks

## Abstract

One-step adsorption separation of C_2_H_4_ from ternary C_2_ hydrocarbon mixtures remains an important and challenging goal for petrochemical industry. Current physisorbents either suffer from unsatisfied separation performance, poor stability, or are difficult to scale up. Herein, we report a strategy of constructing multiple supramolecular binding sites in a robust and scalable MOF (Al-PyDC) for highly efficient one-step C_2_H_4_ purification from ternary mixtures. Owing to suitable pore confinement with multiple supramolecular binding sites, Al-PyDC exhibits one of the highest C_2_H_2_ and C_2_H_6_ uptakes and selectivities over C_2_H_4_ at ambient conditions. The gas binding sites have been visualized by single-crystal X-ray diffraction studies, unveiling that the low-polarity pore surfaces with abundant electronegative N/O sites provide stronger multiple supramolecular interactions with C_2_H_2_ and C_2_H_6_ over C_2_H_4_. Breakthrough experiments showed that polymer-grade C_2_H_4_ can be separated from ternary mixtures with a maximum productivity of 1.61 mmol g^−1^. This material can be prepared from two simple reagents using a green synthesis method with water as the sole solvent, and its synthesis can be easily scaled to multikilogram batches. Al-PyDC achieves an effective combination of benchmark separation performance, high stability/recyclability, green synthesis and easy scalability to address major challenges for industrial one-step C_2_H_4_ purification.

## Abstract (web summary)

One-step separation of C2H4 from ternary C2 hydrocarbon mixtures remains a challenge for current physisorbents. Here, the authors reported a strategy of designing multiple supramolecular binding sites in a robust and scalable MOF for highly efficient one-step C2H4 purification from ternary mixtures.

## Introduction

Purification of olefins such as ethylene (C_2_H_4_) accounts for 0.3% of global energy consumption, highlighting as one of seven most important chemical separations^1^. As the largest-volume product of the chemical industry, the worldwide production of C_2_H_4_ has exceeded 200 million tons per year in 2020 and will continuously increase in the future. At present, polymer-grade C_2_H_4_ is mainly produced by energy-intensive separation of downstream C_2_ hydrocarbon gas mixtures during the steam cracking process^2^. Acetylene (C_2_H_2_) is first removed through catalytic hydrogenation using noble-metal catalysts under high temperatures and pressures, and then ethane (C_2_H_6_) is separated by energy-intensive cryogenic distillation^3^. These high energy footprints associated with C_2_H_4_ purification have pushed the development of cost- and energy-efficient separation technologies to a level of utmost importance.

**Fig. 1:**
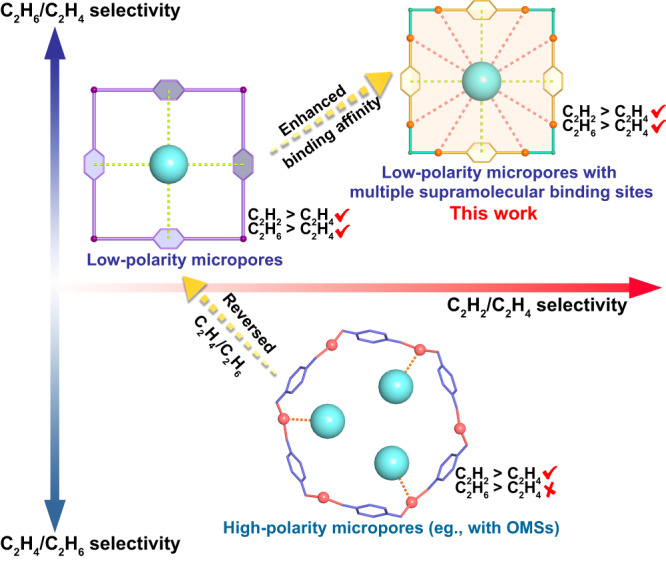
Illustration of strategy. Schematic illustration of the proposed multiple supramolecular binding sites for boosting the selective binding of C_2_H_2_ and C_2_H_6_ over C_2_H_4_ molecule.

Adsorption separation technology based on porous materials has been demonstrated to be a promising technology to replace traditional cryogenic distillation and thus to fulfill the energy-efficient separation economy^4–9^. Highly efficient separation of C_2_H_4_ from binary C_2_H_2_/C_2_H_4_ and C_2_H_6_/C_2_H_4_ mixtures has been well addressed by various materials including metal−organic frameworks (MOFs)^10–19^, covalent organic frameworks (COFs)^20^, zeolites and so on^21–23^. Amongst them, microporous MOFs have shown particular promise for gas separations because of the powerful tunability on pore size and functionality^24–30^. Compared with separation of binary mixtures, simultaneous removal of C_2_H_2_ and C_2_H_6_ from ternary C_2_ mixtures would be more desirable to directly obtain polymer-grade C_2_H_4_, which can simplify the separation process to result in large energy saving. However, the simultaneous separation of C_2_H_2_ and C_2_H_6_ impurities from C_2_H_4_ remains a daunting challenge for classical physisorbents, since all the physical properties of C_2_H_4_ molecule lie between C_2_H_2_ and C_2_H_6_ (Supplementary Table 1)^31, 32^. Owing to the higher quadrupole moment and acidity of C_2_H_2_ over C_2_H_4_, the preferential adsorption of C_2_H_2_ over C_2_H_4_ with high selectivities can be generally achieved by MOFs with highly polar groups (e.g., open metal sites and fluoridated anion pillars)^33–41^; however, these kinds of materials bind more strongly with C_2_H_4_ over C_2_H_6_ to result in the selective adsorption of C_2_H_4_ over C_2_H_6_ (Fig. 1). Given that C_2_H_6_ has a higher polarizability than C_2_H_4_ (44.7 × 10^−25^ vs 42.52 × 10^−25^ cm^3^), the selective adsorption of C_2_H_6_ over C_2_H_4_ is commonly favored by MOFs with nonpolar/inert pore surfaces (e.g., aromatic or aliphatic moieties), wherein soft supramolecular interactions (e.g., C−H···π or hydrogen bonding) make major contributions^42–48^. A handful of C_2_H_6_-selective MOFs have been recently discovered to show the preferential adsorption of both C_2_H_2_ and C_2_H_6_ over C_2_H_4_ via various supramolecular interactions^49–59^. However, the weak nature of supramolecular interactions commonly makes the C_2_H_2_ or C_2_H_6_ binding affinity not so sufficient, resulting in poor gas uptake or insufficient selectivity to preclude most of them from being highly selective (Fig. 1). For example, Azole-Th-1 and Zn(ad)(int) exhibit relatively high C_2_H_6_/C_2_H_4_ selectivity, whereas their C_2_H_2_ uptake and selectivity are quite low because of their insufficient C_2_H_2_ binding affinity^49,54^. UiO-67-(NH_2_)_2_ and CuTiF_6_-TPPY holds the benchmark C_2_H_2_ and C_2_H_6_ uptakes or selectivities, but limited by inadequate gas selectivities or uptakes^53,59^, respectively. Ideal adsorbents for one-step C_2_ separation should possess both high C_2_H_2_ and C_2_H_6_ adsorption capacities and selectivities over C_2_H_4_, which can maximize the productivity and purity of C_2_H_4_ purification. Besides separation performance, some other factors such as stability, economy feasibility, and synthesis scalability, also need to be considered for large-scale industrial applications. However, most of the reported C_2_H_2_/C_2_H_6_-selective MOFs suffer from the drawbacks of poor water stability, high cost, or low scalability, largely impeding their applications in industrial scenarios. Currently, there are no reports existing on kilogram-scale synthesis of MOFs relevant for one-step C_2_H_2_/C_2_H_6_/C_2_H_4_ separation. Developing ideal MOF adsorbents that fully merge high separation performance with high stability, economy feasibility and easy scalability of synthesis has never been achieved yet for this important one-step C_2_H_4_ purification.

**Fig. 2:**
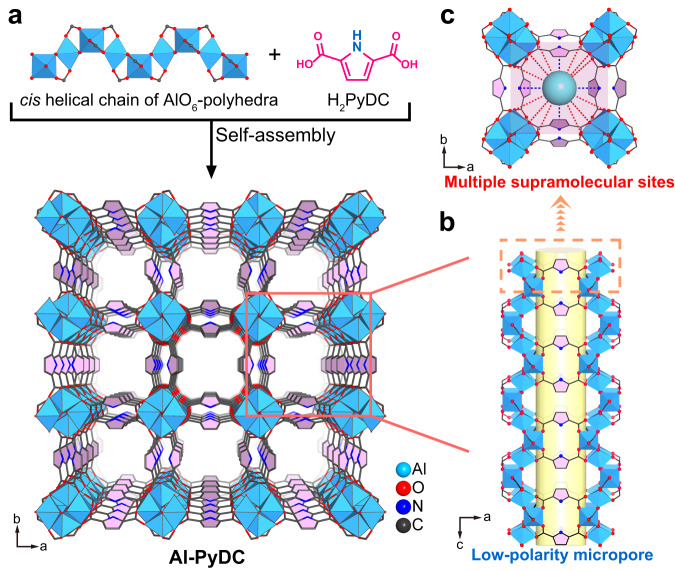
Crystal structure description of the activated Al-PyDC. **a** The framework of Al-PyDC formed by AlO_6_-polyhedra chains and H_2_PyDC linkers. **b** The 1D square channel with low-polarity micropore surfaces, decorated by abundant O/N sites and aromatic rings. **c** Multiple supramolecular binding sites in the pore of Al-PyDC.

To overcome the weakness nature of supramolecular interactions, an effective design strategy is to incorporate multiple supramolecular binding sites into MOFs with suitable pore sizes to enforce C_2_H_2_ and C_2_H_6_ binding affinity (Fig. 1), thus concurrently improving their uptake and selectivity over C_2_H_4_. Recent studies have shown that the incorporation of electronegative oxygen or nitrogen sites into MOFs can provide hydrogen-bonding interactions with acidic C_2_H_2_, and thus enhance C_2_H_2_ adsorption and separation capacities^59–63^. Further, such O/N binding sites may also enable the formation of more numbers of supramolecular interactions with C_2_H_6_ molecule than with C_2_H_4_, given that C_2_H_6_ has larger molecule size and more H atoms^16, 64^. With the above considerations in mind, we herein report a strategy of designing multiple supramolecular binding sites in microporous MOFs for highly efficient one-step separation of C_2_H_4_ from ternary C_2_ mixtures. We target this matter in a known and robust Al-based MOF, [Al(OH)PyDC]_n_ (Al-PyDC, also called KMF-1 or MOF-313, H_2_PyDC = 2,5-pyrroledicarboxylate)^65,66^. This material consists of cis-corner-sharing octahedra AlO_6_ chains linked by N-containing aromatic ligands to form one-dimensional pore channels with a suitable size of 5.8 Å, wherein a large number of polar O sites and N-heterocyclic rings are densely arranged along pore channels to provide abundant supramolecular binding sites (Fig. 2). Al-PyDC thus exhibits one of the highest C_2_H_2_ and C_2_H_6_ uptakes (8.24 and 4.20 mmol g^−1^) and selectivities (4.3 and 1.9) over C_2_H_4_ at ambient conditions, outperforming most benchmark materials reported so far. Single-crystal X-ray diffraction (SCXRD) studies on gas-loaded Al-PyDC for all the C_2_ molecules visually unveiled that multiple O/N sites on channel-pore surfaces provide stronger multiple supramolecular interactions with both C_2_H_2_ and C_2_H_6_ over C_2_H_4_, accounting for both very high C_2_H_2_ and C_2_H_6_ uptakes and selectivities. Dynamic breakthrough experiments confirm its exceptional one-step C_2_H_4_ purification from 1/49.5/49.5 or 1/9/90 C_2_H_2_/C_2_H_6_/C_2_H_4_ mixtures at ambient conditions, affording the maximal polymer-grade C_2_H_4_ productivity of 0.66 or 1.61 mmol g^−1^. Most importantly, Al-PyDC was synthesized from two simple and commercially available reagents H_2_PyDC and AlCl_3_·6H_2_O using water as the sole solvent, and we showed that its synthesis can be easily scaled-up to multikilogram batches in a high yield of 92% at mild and water-based conditions. The production of Al-PyDC can be considered as a scalable green synthesis. The combined advantages of benchmark separation performance, high stability/recyclability, green synthesis and easy scalability make it as a benchmark material for industrial one-step C_2_H_2_/C_2_H_6_/C_2_H_4_ separation.

## Results

### Synthesis and structural characterization

The Al-PyDC samples were readily synthesized as microcrystalline powders by reaction of the simple dicarboxylate ligand (H_2_PyDC) and AlCl_3_·6H_2_O according to the previous literature with procedural optimization (see the Method for details)^65^. The crystal structures of the as-synthesized and activated Al-PyDC have been determined by Chang et al. based on the synchrotron powder diffraction data^65^. In spite of this, it is still highly important to obtain large enough single crystals of Al-PyDC for SCXRD analysis, which facilitates to not only check the accurate structure information but also determine gas-loaded crystal structures to visually identify the binding sites of C_2_ molecules. While it is very challenging to get large single crystals for Al-MOFs, we here successfully prepared large colorless square-block-shaped crystals for Al-PyDC through great endeavors to optimize the growth method (Supplementary Fig. 1). The SCXRD analysis revealed that hydrated Al-PyDC crystallizes in a non-centrosymmetric tetragonal space group (*I*4_1_md) with unit cell parameters of *a* = *b* = 21.1895(3) Å and *c* = 10.6283(3) Å, which are consistent with the results obtained by synchrotron powder diffraction data. After removing all the guest molecules, the activated structure of Al-PyDC can transfer to a new tetragonal symmetry of *I*4_1_/amd, as evidenced by the literature and our following gas-loaded crystal structures (Supplementary Table 4).

The crystal structure of the activated Al-PyDC is depicted in Fig. 2. Each octahedrally coordinated Al-centre is linked to four oxygen atoms from four PyDC linkers and two *cis* bridging OH^−^ groups. The octahedral AlO_6_-polyhedras form one-dimensional (1D) four-fold helical chains via corner sharing, which are further interconnected with each other by the PyDC linkers to result in a 3D framework (Fig. 2a). As shown in Fig. 2b, Al-PyDC exhibits a 1D square-shaped pore channel with a pore window size of 5.8 Å in diameter along the *c* axis. This aperture size is larger than the kinetic diameter of all C_2_ molecules so as to favor the rapid diffusion of these gas molecules. Due to the full coordination of the Al-centre, there are no polar open metal sites (OMSs) existed in the framework of Al-PyDC. Most importantly, the surface of pore channels is surrounded by abundant naked oxygen atoms and N-containing aromatic rings originated from organic linkers and bridging OH^−^ groups. Such nonpolar pore surface decorated by abundant O/N sites and aromatic rings may provide multiple supramolecular binding sites to preferentially interact with C_2_H_6_ than with C_2_H_4_ molecule (Fig. 2c). Further, recent studies on Al-MOFs have shown that the densely distributed oxygen atoms around the channels can provide high-density H-bonding interactions with C_2_H_2_ molecule, leading to high C_2_H_2_ uptakes and selectivities^61,62^. Therefore, we reasoned that such multiple supramolecular binding sites within OMS-free Al-PyDC may show the great potential to not only concurrently reinforce C_2_H_2_ and C_2_H_6_ binding affinity for high gas uptakes, but also provide more preferential adsorption of C_2_H_2_ and C_2_H_6_ over C_2_H_4_ for high selectivities.

### Gas adsorption measurements

**Fig. 3:**
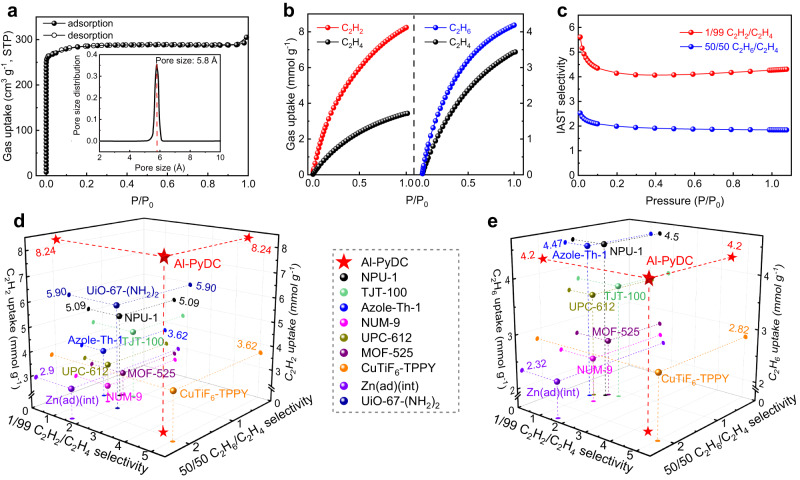
Gas sorption properties. **a** N_2_ sorption isotherms of Al-PyDC at 77 K. Inset shows pore size distribution of Al-PyDC calculated based on NLDFT model. **b** Adsorption isotherms of Al-PyDC for C_2_H_2_, C_2_H_6_ and C_2_H_4_ at 296 K. **c** Predicted IAST selectivity curves for C_2_H_2_/C_2_H_4_ and C_2_H_6_/C_2_H_4_ mixtures at 296 K. **d** Comparison of C_2_H_2_ uptake and gas selectivities for Al-PyDC with the promising C_2_H_2_/C_2_H_6_-selective materials at ambient conditions. **e** Comparison of C_2_H_6_ uptake and gas selectivities for Al-PyDC with the indicated materials.

The solvent-exchanged Al-PyDC sample was evacuated at room temperature for 12 h and then 393 K for 12 h until the outgas rate was 5 μmHg min^−1^, affording the fully activated material (Supplementary Fig. 2). The permanent porosity of the activated Al-PyDC was determined by nitrogen (N_2_) adsorption isotherms at 77 K. As shown in Fig. 3a, Al-PyDC takes up a 305 cm^3^ g^−1^ amount of N_2_ at 77 K and 1 bar, with a significant type I sorption behavior. The Brunauer−Emmett−Teller surface area and pore volume were calculated to be 1134 m^2^ g^−1^ and 0.472 cm^3^ g^−1^ (Supplementary Fig. 3), in good agreement with the values (1130 m^2^ g^−1^ and 0.473 cm^3^ g^−1^) reported in the literature^65^. The pore size distribution, determined by Non-Local Density Functional Theory (NLDFT) method based on 77 K N_2_ isotherms, shows a moderate pore size of 5.8 Å (Fig. 3a), which is consistent well with the value obtained from the crystal structure.

Pure-component adsorption isotherms of C_2_ hydrocarbons for Al-PyDC were measured at 273, 296, and 313 K up to 1 bar (Fig. 3b, Supplementary Figs. 4 and 5), respectively. Owing to the OMS-free and nonpolar pore surfaces, Al-PyDC exhibits an obviously preferential adsorption of C_2_H_2_ and C_2_H_6_ over C_2_H_4_ at 296 K (Fig. 3b). The C_2_ gas uptakes at 296 K and 1 bar follow the expected sequence of C_2_H_2_ (8.24 mmol g^−1^) > C_2_H_6_ (4.20 mmol g^−1^) > C_2_H_4_ (3.44 mmol g^−1^). This endows Al-PyDC with a promising capacity for efficient one-step separation of C_2_H_4_ from ternary mixtures. It is worthy to note that the C_2_H_2_ uptake (8.24 mmol g^−1^) at 1 bar and 296 K is the highest among all the C_2_H_2_/C_2_H_6_-selective MOFs reported so far (Fig. 3d and Supplementary Fig. 6), notably larger than that of most benchmark materials such as CuTiF_6_-TPPY (3.62 mmol g^−1^)^53^, TJT-100 (4.46 mmol g^−1^)^55^, NPU-1 (5.09 mmol g^−1^)^56^ and UiO-67-NH_2_ (5.90 mmol g^−1^)^59^. Further, the C_2_H_6_ uptake of Al-PyDC is also among the top-tier values for the relevant MOFs (Fig. 3e), surpassing that of Ag-PCM-102 (3.70 mmol g^−1^)^52^, CuTiF_6_-TPPY (2.82 mmol g^−1^)^53^, TJT-100 (3.75 mmol g^−1^)^55^ and comparable to Azole-Th-1 (4.47 mmol g^−1^)^49^ and NPU-1 (4.50 mmol g^−1^)^56^. Evidently, both the top-tier C_2_H_2_ and C_2_H_6_ uptakes make Al-PyDC as one of the best materials for ternary C_2_ separation. Further, we also investigated the time-dependent ads/desorption kinetics profiles of Al-PyDC at 296 K. As shown in Supplementary Fig. 7, Al-PyDC exhibits fast adsorption kinetics for all the C_2_ molecules and the adsorbed molecules can be completely removed under a high vacuum quickly, mainly attributed to its comparably large pore channels.

These adsorption discrepancies between C_2_ hydrocarbons can be partially explained by the experimental isosteric heat of adsorption (*Q*_st_), calculated by adsorption isotherms at different temperatures (Supplementary Figs. 9−11). As shown in Supplementary Fig. 12, the calculated *Q*_st_ values at zero loading were determined to follow the order of C_2_H_2_ (35.3 kJ mol^−1^) > C_2_H_6_ (30.1 kJ mol^−1^) > C_2_H_4_ (27.8 kJ mol^−1^). Such higher *Q*_st_ values for C_2_H_2_ and C_2_H_6_ compared with C_2_H_4_ confirm the stronger binding affinities of Al-PyDC with the former two gases, which is consistent well with that found in C_2_ gas uptakes. Compared to most reported C_2_H_2_/C_2_H_6_-selective materials, Al-PyDC also shows relatively higher *Q*_st_ values for C_2_H_2_ and C_2_H_6_ (Supplementary Fig. 13), probably attributed to the multiple supramolecular binding sites observed in Al-PyDC.

Ideal Adsorbed Solution Theory (IAST) was utilized to estimate the adsorptive selectivity of Al-PyDC for both 1/99 C_2_H_2_/C_2_H_4_ and 50/50 C_2_H_6_/C_2_H_4_ mixtures. As shown in Fig. 3c, Al-PyDC exhibits both high C_2_H_2_/C_2_H_4_ selectivity of 4.3 and C_2_H_6_/C_2_H_4_ selectivity of 1.9 at 296 K and 1 bar. The C_2_H_2_/C_2_H_4_ selectivity is the highest among all of the reported C_2_H_2_/C_2_H_6_-selective MOFs except CuTiF_6_-TPPY^53^, significantly larger than that of some best-performing materials including Zn(ad)(int) (1.61)^54^, TJT-100 (1.8)^55^, and NPU-1 (1.4)^56^ and UiO-67-NH_2_ (2.1)^59^. Furthermore, the C_2_H_6_/C_2_H_4_ selectivity of Al-PyDC is also among the highest for the existing C_2_H_2_/C_2_H_6_-selective MOFs, outperforming Azole-Th-1 (1.46)^49^, TJT-100 (1.8)^55^, UiO-67-NH_2_ (1.7)^59^ and other benchmark materials. As shown in Figs. 3d, e, when we set the uptakes and selectivities as concurrent objectives for both C_2_H_2_/C_2_H_4_ and C_2_H_6_/C_2_H_4_ separations, Al-PyDC exhibits by far the best combination of very high uptakes and selectivities toward both separations, making it as the current benchmark for one-step separation of C_2_H_4_ from ternary C_2_ mixtures.

### Binding-site determination

**Fig. 4:**
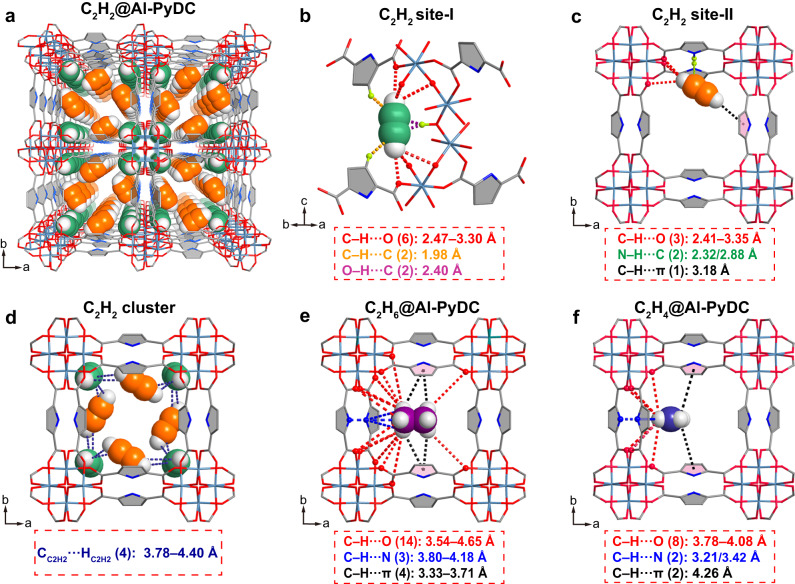
Adsorption binding sites of C_2_H_2_, C_2_H_6_ and C_2_H_4_ in Al-PyDC. **a** The SCXRD structure of C_2_H_2_-loaded Al-PyDC viewed along the *c* axis, indicating two types of C_2_H_2_ binding sites. **b** Illustration of C_2_H_2_ binding site-I, (**c)** C_2_H_2_ binding site-II, and (**d**) dense packing of C_2_H_2_ molecules within Al-PyDC. **e** The C_2_H_6_ binding site and (**f**) C_2_H_4_ binding site in gas-loaded Al-PyDC, determined by SCXRD analysis.

To visualize the locations of all C_2_ molecules and thus elucidate the origin of the observed higher C_2_H_6_ and C_2_H_2_ uptakes over C_2_H_4_, we carried out the in situ SCXRD experiments on gas-loaded Al-PyDC crystals. The SCXRD data were collected at 200 K and 1 bar. According to the C_2_H_2_-loaded SCXRD analysis, Al-PyDC was found to exhibit two types of binding sites (site-I and site-II) for the adsorbed C_2_H_2_ molecules (Fig. 4a). As shown in Fig. 4b, the adsorbed C_2_H_2_ molecule in site-I is mainly located at the corner of square-shaped pore channel. This C_2_H_2_ molecule exhibits multiple interactions with six carboxylate oxygen atoms through six C−H···O hydrogen bonds with the distances of 2.47–3.30 Å. In addition, C_2_H_2_ molecule also interacts with μ-OH site of the Al-octahedral chain through two supramolecular interactions (O−H···C = 1.98 Å), and with two C − H groups from surrounding pyrrole rings through weak supramolecular interactions (C_C2H2_···H_pyrrole_ = 2.40 Å). The C_2_H_2_ binding site-II was found to be located around the channel wall and in close proximity to the PyDC linker (Fig. 4c). The site-II acetylene molecule interacts with three carboxylate oxygen atoms through C−H···O hydrogen bonds (2.41–3.35 Å) and with one pyrrole ring to form the van der Waals (vdW) interaction (C−H···π = 3.18 Å). Further, the C_2_H_2_ molecule also binds with two N−H groups from two PyDC linkers through weak supramolecular interactions (N−H···C_C2H2_ = 2.32/2.88 Å). Such multiple supramolecular interactions in site-I and site-II were found to cooperatively interact with the adsorbed C_2_H_2_ molecules. Most importantly, as shown in Fig. 4d and Supplementary Fig. 14, due to the dense distribution of these two types of binding sites within the channels, each adsorbed C_2_H_2_ molecule in site-I interacts with two adjacent C_2_H_2_ molecules in site-II through four C−H···C−H interactions (3.78–4.40 Å). Such cooperative interactions between guest molecules enable the dense packing of C_2_H_2_ molecules inside the pore channels, resulting in the ultrahigh C_2_H_2_ uptake capacity of Al-PyDC. Full occupation of these adsorption sites corresponds to 10.1 mmol g^−1^ gas uptake, which is close to the saturated C_2_H_2_ uptake (11.1 mmol g^−1^) at 196 K (Supplementary Fig. 15). This also implies that about 81.6% of these C_2_H_2_ adsorption sites are occupied at 296 K and 1 bar.

For C_2_H_6_ molecule, the adsorption location is approximately the same as that of C_2_H_2_ binding site-II. Due to its larger molecular size, C_2_H_6_ adopts an adsorption orientation different from that of site-II C_2_H_2_ molecule because of the steric restriction. The C_2_H_6_ orientation is perpendicular to the PyDC linker axis. As shown in Fig. 4e, multiple supramolecular interactions were observed between the adsorbed C_2_H_6_ molecule and the host framework. Each C_2_H_6_ molecule is H-bonded to twelve carboxylate O atoms around the pore through fourteen C−H···O interactions with the distances of 3.54–4.65 Å. In addition, C_2_H_6_ molecule also interacts with two pyrrole N atoms through three C−H···N interactions (3.80–4.18 Å) and with pyrrole ring on both sides through four C−H···π interactions (3.33–3.71 Å). In comparison, the adsorbed C_2_H_4_ molecules are located at the similar positions in the square-shaped channels. As depicted in Fig. 4f, each C_2_H_4_ molecule interacts with carboxylate O atoms to form only six C−H···O H-bonds with the distances of 3.83–4.08 Å, with two pyrrole N atoms through C–H···N interactions (3.21 and 3.42 Å) and with pyrrole ring on both sides through two C−H···π interactions (4.26 Å). Evidently, due to the more number of H atoms and larger molecular size of C_2_H_6_, the adsorbed C_2_H_6_ molecule exhibits much more number of weak supramolecular interactions with the framework, leading to the stronger binding affinity than C_2_H_4_ molecule. This can be further confirmed by the higher experimental binding energies of C_2_H_6_ (30.1 kJ mol^−1^) than C_2_H_4_ (27.8 kJ mol^−1^) observed in Al-PyDC. All of the above results can visually elucidate the adsorption and separation phenomenon on C_2_ gas mixtures.

### Dynamic breakthrough studies

**Fig. 5:**
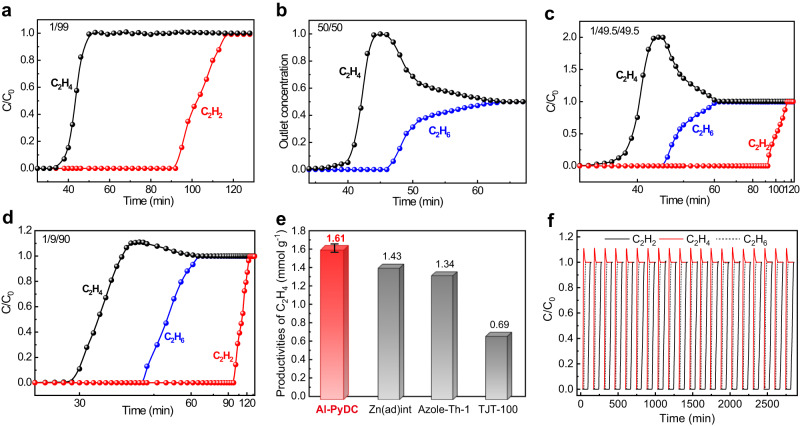
Dynamic breakthrough experiments. Experimental column breakthrough curves of Al-PyDC for (**a**) C_2_H_2_/C_2_H_4_ (1/99), (**b**) C_2_H_6_/C_2_H_4_ (50/50) and (**c**) C_2_H_2_/C_2_H_6_/C_2_H_4_ (1/49.5/49.5) mixtures under ambient conditions. **d** Experimental column breakthrough curves of Al-PyDC for C_2_H_2_/C_2_H_6_/C_2_H_4_ (1/9/90) mixtures under ambient conditions. **e** Comparison of pure C_2_H_4_ productivity for Al-PyDC (the averaged value was obtained from five independent tests, and the error bar is the standard deviation) with the top-performing materials reported. **f** Twenty separation cycles of breakthrough experiments on Al-PyDC for C_2_H_2_/C_2_H_6_/C_2_H_4_ (1/9/90) mixture.

Dynamic breakthrough experiments were first conducted on a packed column filled with activated Al-PyDC to evaluate the separation performance for binary C_2_H_6_/C_2_H_4_ (50/50) and C_2_H_2_/C_2_H_4_ (1/99) mixtures under a gas flow of 1.25 mL min^−1^ at 296 K. As shown in Fig. 5a, owing to the ultrahigh C_2_H_2_ uptake and large C_2_H_2_/C_2_H_4_ selectivity, Al-PyDC exhibits a highly efficient separation capacity for 1/99 C_2_H_2_/C_2_H_4_ mixture, wherein C_2_H_4_ gas first eluted through the adsorption bed at 34 min, while C_2_H_2_ breakthrough did not occur until 92 min. During this time interval, pure C_2_H_4_ production (> 99.999%) from the outlet effluent for a given cycle was calculated to be 7.93 mmol g^−1^. This C_2_H_4_ productivity is even much higher than some top-performing materials for single C_2_H_2_/C_2_H_4_ separation, such as UTSA-100a (1.13 mmol g^−1^)^11^ and SIFSIX-3-Zn (1.94 mmol g^−1^)^37^. Further, the efficient separation of C_2_H_4_ from C_2_H_6_/C_2_H_4_ mixture can be also accomplished by Al-PyDC (Fig. 5b), with a high pure C_2_H_4_ productivity of 0.68 mmol g^−1^ at the outlet. This productivity is higher than some promising C_2_H_6_-selective materials such as Cu(Qc)_2_ (0.42 mmol g^−1^)^42^, MAF-49 (0.28 mmol g^−1^)^65^ and even comparable to the benchmark Fe_2_(O_2_)(dobdc)_2_ (0.79 mmol g^−1^)^14^.

Next, we examined the separation performance of Al-PyDC for actual C_2_H_2_/C_2_H_6_/C_2_H_4_ ternary mixtures at the same conditions. Figure 5c reveals that complete separation of both C_2_H_6_ and C_2_H_2_ from 3-component C_2_H_2_/C_2_H_6_/C_2_H_4_ (1/49.5/49.5) mixture can be fulfilled by Al-PyDC. It was found that C_2_H_4_ was first eluted at 33 min to yield a pure gas with an undetectable amount of C_2_H_6_ and C_2_H_2_ (the detection limit of the instrument is 0.01%). After that, C_2_H_6_ gas secondly passed through the adsorption bed at 46 min, and the adsorbent retained C_2_H_2_ lastly until 92 min. During the time interval between C_2_H_4_ and C_2_H_6_/C_2_H_2_ breakthrough, pure C_2_H_4_ productivity of Al-PyDC from the outlet effluent for a given cycle was calculated up to 0.66 mmol g^−1^. This C_2_H_4_ productivity is even higher than the previously benchmark UiO-67-(NH_2_)_2_ (0.55 mmol g^−1^)^59^. Given the fact that the C_2_ fraction obtained from cracked gas sometimes contains a small portion of C_2_H_6_ (ca. 6–10%), we further evaluated its separation capacity on a ternary C_2_H_2_/C_2_H_6_/C_2_H_4_ (1/9/90) mixture at the same conditions. As shown in Fig. 5d, the much later breakthrough times of both C_2_H_2_ and C_2_H_6_ (100 and 43 min) than that of C_2_H_4_ (29 min) reveal that simultaneous removal of C_2_H_2_ and C_2_H_6_ can be fulfilled by Al-PyDC. The productivity of pure C_2_H_4_ (over 99.9%) from the outlet effluent was calculated up to 1.61 mmol g^−1^ (Fig. 5e), which is the highest among the reported best-performing C_2_H_2_/C_2_H_6_-selective materials including Azole-Th-1 (1.34 mmol g^−1^, 1/9/90)^49^, Zn(ad)(int) (1.43 mmol g^−1^, 1/10/89)^54^ and TJT-100 (0.69 mmol g^−1^, 0.5/0.5/99)^55^. The highly efficient separation capacity can be still retained when the flow rate of the mixed gases accelerated to 5.0 and 10.0 mL min^−1^ (Supplementary Fig. 17).

For practical applications, the adsorbent should possess good recyclability and structural stability. To test the recyclability of Al-PyDC, we firstly carried out cycling experiments on Al-PyDC for single-component C_2_H_2_ or C_2_H_6_ adsorption at 1 bar and 296 K, followed by desorption under vacuum at room temperature. As shown in Supplementary Fig. 18, the experimental cycling results indicate that there was no noticeable loss in C_2_H_2_ and C_2_H_6_ adsorption capacities for Al-PyDC over 30 cycles. Next, breakthrough separation experiments on both C_2_H_2_/C_2_H_6_/C_2_H_4_ (1/49.5/49.5) and C_2_H_2_/C_2_H_6_/C_2_H_4_ (1/9/90) mixtures were cycled numerous times to further assess the recyclability of Al-PyDC (Fig. 5f and Supplementary Figs. 19−21), wherein the sample was fully regenerated between cycles under sweeping He gas at 373 K. The cycling results show that there was no obvious loss in the separation capacity for Al-PyDC over 20 cycles, confirming its good recyclability for this separation. Given that the actual feed streams typically contain small amount of acidic gases (e.g., H_2_S),^67^ the breakthrough experiments for 1/9/90 C_2_H_2_/C_2_H_6_/C_2_H_4_ mixture with 1000 ppm H_2_S were performed on Al-PyDC to investigate the effect of acidic H_2_S gas on separation performance. As shown in Supplementary Fig. 22, the separation performance of Al-PyDC remains almost unchanged to afford the comparable C_2_H_4_ productivity of 1.57 mmol g^−1^, and the separation capacity can be preserved over six continuous cycles under the presence of acid gases. These results have demonstrated that Al-PyDC can be recycled for repeated separation cycles without any loss of performance even under the presence of acid gases.

### Material stability and scale-up synthesis

**Fig. 6:**
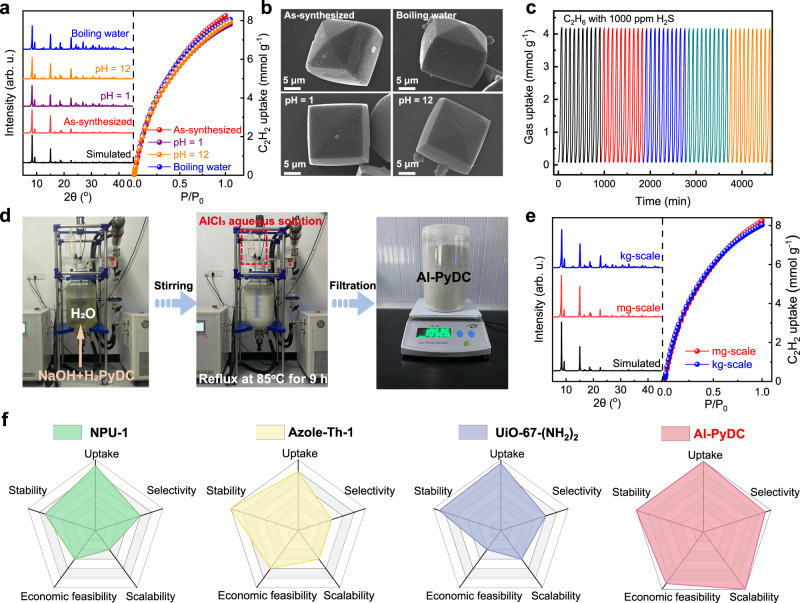
Stability and scalable synthesis. **a** PXRD patterns and C_2_H_2_ adsorption isotherms and (**b**) SEM images of Al-PyDC samples after treatment with different conditions. **c** Gas adsorption on Al-PyDC over 50 consecutive adsorption-desorption cycles for C_2_H_6_ gas containing 1000 ppm H_2_S at 296 K, in which the sample was regenerated by N_2_ sweeping at 373 K. **d** The kilogram-scale synthesis of Al-PyDC by a green and facile method. **e** PXRD patterns and C_2_H_2_ adsorption isotherms of Al-PyDC samples obtained from various-scale synthesis. **f** The comprehensive comparison of separation performance, stability, economic feasibility and scalability for Al-PyDC and other benchmark materials.

Besides separation performance, material stability and scale-up synthesis are two most important concerns for industrial applications. We first examined the chemical stability of Al-PyDC after treatment under different conditions, monitored by powder X-ray diffraction (PXRD) and gas adsorption measurements. As shown in Fig. 6a, after immersion in water, boiling water, and aqueous solutions of pH 1 and 12 for 3 days, the PXRD studies revealed that the framework of Al-PyDC can retain its structural integrity without any phase change and loss of crystallinity observed. Such ultrahigh chemical stability was also confirmed by N_2_ adsorption isotherms at 77 K and C_2_H_2_/C_2_H_6_ uptake capacities at 296 K after different treatment, wherein all the gas adsorption amounts show no obvious decrease compared to those of the pristine sample (Supplementary Fig. 23). In addition, scanning electron microscope (SEM) and optical images of Al-PyDC crystals showed that there are no obvious changes observed in their morphology and surface after the treatment with water and pH solutions (Fig. 6b and Supplementary Fig. 24). The variable temperature PXRD patterns indicate that Al-PyDC is thermally stable up to 350 °C (Supplementary Fig. 25). Therefore, this material shows one of the best chemical and thermal stabilities among the reported MOFs (Supplementary Table 8), even comparable to some representative stable MOFs including MIL-101(Cr), BUT-12 and PCN-250.^68–70^ Considering that the actual working environment would contain trace amount of acid gases in feed streams^67^, we further assess the stability of Al-PyDC under acid gas circumstances. We first carried out the repeated adsorption/desorption cycling experiments for C_2_H_6_ gas containing 1000 ppm acidic H_2_S gas, in which the sample was regenerated by N_2_ sweeping at 373 K. As shown in Fig. 6c, the C_2_H_6_ adsorption capacity can be maintained with no noticeable loss after 50 cycles in the presence of acidic H_2_S. Further, the breakthrough cycling experiments on C_2_H_2_/C_2_H_6_/C_2_H_4_ mixtures containing 1000 ppm H_2_S further confirmed that the separation capacity of Al-PyDC can be preserved without any loss of performance over six cycles under the presence of acid gases (Supplementary Fig. 22). The PXRD pattern and 77 K N_2_ sorption isotherms after repeated breakthrough cycles indicate that Al-PyDC can maintain its structural integrity (Supplementary Fig. 26). These results confirm its high structural stability under practical circumstances.

With respect to large-scale practical applications, it is crucial that the scale-up synthesis of MOFs should be viable from a technical and economic point of view. The cost analysis for various MOF syntheses indicates that the raw material costs are often prohibitively high, especially for organic linkers or using organic solvents as reaction solutions. Further, synthetic conditions can also have an important influence on the economics. For instance, the necessary use of high-temperature or high-pressure apparatus for MOF syntheses would be not only costly but also bring high expenses on safety precautions. Al-PyDC exhibits none of these disadvantageous conditions. The reported literature showed that Al-PyDC can be prepared from simple and commercially available reagents of H_2_PyDC and Al_2_(SO_4_)_3_·18H_2_O in water solvent at a temperature of 120 °C^65^. However, the synthetic temperature (120 °C) is still much higher than the water boiling point, which is detrimental to the scale-up synthesis. To reduce the synthetic temperature, we here optimized the reaction conditions by changing the base equivalents and aluminum salt (see the Method for details). Our experiments revealed that Al-PyDC can be readily synthesized from H_2_PyDC and AlCl_3_·6H_2_O in water solvent at a much lower temperature (85 °C). These mild and water-based conditions can be considered as a scalable green synthesis, which are particularly advantageous from safety and environmental aspects. As shown in Fig. 6d and Supplementary Fig. 27, the kilogram-scale production of Al-PyDC sample was easily performed by using a reflux-based synthesis method in a 30 L reaction vessel. This protocol provided more than 1.0 kg of as-synthesized Al-PyDC per reaction batch in a high yield of 92% within 9 h, resulting in an exceptional space-time yield (STY) over 126 kg m^−3^ day^−1^. For a comparison, the STYs of zeolites are commonly in the range of 50–150 kg m^−3^ day^−1^^12^. The Al-PyDC products synthesized at this large-scale exhibit similar crystallinity and gas uptake capacities compared to the material produced at a small scale, as verified by PXRD analysis and gas adsorption isotherms (Fig. 6e and Supplementary Fig. 29). Further, dynamic breakthrough experiments on activated Al-PyDC sample synthesized in this low temperature procedure showed the same separation capacity for ternary C_2_ hydrocarbon mixtures (Supplementary Fig. 29), without any loss in C_2_H_4_ productivity.

A perfect adsorbent for industrial one-step C_2_H_4_ purification from ternary mixtures should meet the following criteria: (1) large C_2_H_2_ and C_2_H_6_ adsorption capacities; (2) high C_2_H_2_/C_2_H_4_ and C_2_H_6_/C_2_H_4_ selectivities; (3) viable chemical/thermal stability; (4) economic feasibility; (5) easy scalability of production. As shown in Fig. 6f, we comprehensively compare all the above criteria between Al-PyDC and other benchmark materials. We have shown that Al-PyDC can meet all of these criteria. In comparison, other benchmark MOFs have better reported properties in one or more of the above-mentioned criteria, but not in all of them. For instance, several benchmark MOFs (e.g., Azole-Th-1, NPU-1, and UiO-67-NH_2_) show highly efficient separation performance for one-step C_2_H_4_ purification; however, they suffer from either the use of toxic organic solvents (e.g., dimethylformamide) and harsh synthesis conditions, or contain expensive and complicated organic linkers (Supplementary Table 8 and Supplementary Fig. 30). These drawbacks lead to an extremely high difficulty on the scale-up production of these materials, making most of them unfavorable for large-scale industrial applications. By far, there are no reports existed on kilogram-scale synthesis of MOFs relevant for one-step C_2_H_2_/C_2_H_6_/C_2_H_4_ separation. With Al-PyDC, we demonstrated that a high-yielding (>90%) and scalable (~1.05 kg) synthesis from simple and commercially available reagents was achieved in water solution and with a high STY value, making the cost of Al-PyDC synthesis more affordable. In terms of separation performance, Al-PyDC exhibits one of the highest C_2_H_2_ and C_2_H_6_ uptakes and selectivities over C_2_H_4_ at ambient conditions, providing a maximum pure C_2_H_4_ productivity for ternary mixtures. Overall, the combined superiorities of green synthesis method, benchmark separation performance, high stability, economic feasibility and easy scalability of production make this material as a promising adsorbent to address the challenges for industrial one-step C_2_H_4_ purification from ternary mixtures.

## Discussion

In summary, we have proposed and demonstrated a scalable and robust Al-MOF (Al-PyDC) with multiple supramolecular binding sites for highly efficient one-step C_2_H_4_ purification from ternary C_2_ mixtures. The gas-loaded SCXRD studies of Al-PyDC visually identified that the low-polarity pore surfaces with abundant O/N sites provide stronger multiple supramolecular interactions with C_2_H_2_ and C_2_H_6_ over C_2_H_4_, thus simultaneously optimizing the C_2_H_2_ and C_2_H_6_ adsorption uptakes and selectivities. This material thereby achieves the top-tier C_2_H_2_ and C_2_H_6_ uptakes (8.24 and 4.20 mmol g^−1^) and selectivities (4.3 and 1.9) over C_2_H_4_ at ambient conditions, outperforming most of the benchmark materials reported to date. The breakthrough experiments affirmed that Al-PyDC can simultaneously separate C_2_H_2_ and C_2_H_6_ from ternary C_2_H_2_/C_2_H_6_/C_2_H_4_ mixtures, affording the record high polymer-grade C_2_H_4_ productivity of 1.61 mmol g^−1^. Most remarkably, Al-PyDC is highly water/pH stable and can be easily produced at kilogram-scale using a green synthesis method. The comprehensive features of high separation performance, notable stability/recyclability, economic feasibility and easy scalability of synthesis make this material as the current benchmark for industrial one-step C_2_H_4_ purification applications. This work also provides an effective approach of designing multiple supramolecular binding sites in microporous MOFs to concurrently enforce C_2_H_2_ and C_2_H_6_ binding affinity for boosting this important gas separation.

## Methods

### Materials

Aluminum chloride hexahydrate (AlCl_3_·6H_2_O, CAS: 231-208-1, purity 99.9%) was purchased from Aladdin, 1H-Pyrrole-2,5-dicarboxylic acid (H_2_PyDC, CAS: 937-27-9, purity 97%) was purchased from Bidepharm (China). N_2_ (99.999%), C_2_H_4_ (99.9%), C_2_H_6_ (99.99%), C_2_H_2_ (99.6%), He (99.999%) and mixed gases of C_2_H_2_/C_2_H_4_ (1/99, v/v), C_2_H_6_/C_2_H_4_ (50/50), C_2_H_2_/C_2_H_6_/C_2_H_4_ (1/49.5/49.5) and C_2_H_2_/C_2_H_6_/C_2_H_4_ (1/9/90) were purchased from JinGong Company (China). All chemicals were used without further purification.

### Synthesis of Al-PyDC powder

The Al-PyDC powder sample was synthesized based on the method reported in the previous literature with modification^65^. H_2_PyDC (310 mg, 2 mmol) and NaOH (240 mg, 6 mmol) were dissolved in H_2_O (6 mL) by sonication for 10 min to obtain clear solution. Afterwards, aqueous AlCl_3_ solution (1 M, 2 mL) was added. The reaction mixture was then kept at 85 °C under refluxed condition for 9 h. The resulting white precipitate of Al-PyDC was separated by using filtration and washed with H_2_O and ethanol.

### Crystallization of Al-PyDC

Single crystals were obtained by fully dissolving H_2_PyDC (39 mg, 0.25 mmol) in aqueous NaOH solution (0.05 M, 5 mL). Afterwards, aqueous AlCl_3_ solution (0.05 M, 5 mL) was slowly added. The resulting solution was incubated for 48 h in a pre-heated oven at 100 °C.

### Scalable synthesis of Al-PyDC

H_2_PyDC (0.9 kg, 5.7 mol) and NaOH (0.684 kg, 17.1 mol) were dissolved in deionized water (17 L) at room temperature with stirring for 30 min to obtain clear solution. Afterwards, AlCl_3_·6H_2_O (1.374 kg, 5.7 mol) was dissolved in 3 L deionized water and transferred to a glass material-feeding funnel. The AlCl_3_ aqueous solution was added at a rate of 3 L per hour to the reaction vessel, resulting in the formation of white precipitate. The solution was then heated and kept at 85 °C for 9 h under reflux condition with the spinner rotating at 150 rpm. After that, the solid product was collected in a 20 L filtration funnel and thoroughly washed with deionized water and ethanol, then vacuum dried at 393 K overnight to obtain ~1.05 kg product with a yield of 92%.

### Sample characterization

The SEM images were obtained from a Hitachi S4800 field-emission scanning electron microscopy (FE-SEM). The microscopy images were captured on an Olympus IX 71 inverted fluorescent microscope. The PXRD patterns in the 2θ = 2–45° range were measured on an X’Pert PRO diffractometer at room temperature using a Cu-Kα (*λ* = 1.54184 Å) radiation source. Thermogravimetric analysis (TGA) was measured on a TA SDT-650 instrument and the sample was heated under N_2_ flow (50 mL min^−1^) with a heating rate of 5 K min^−1^.

### Gas sorption measurements

Before the test, the as-synthesized sample was solvent-exchanged with high-purity methanol over eight times within 3 days to the complete removal of guest solvent molecules from the framework. The solvent-exchanged sample was degassed for 12 h at room temperature and then for another 12 h at 393 K until the outgas rate was 5 μmHg min^−1^. Gas sorption isotherms of C_2_H_2_, C_2_H_6,_ and C_2_H_4_ were obtained from a Micromeritics ASAP 2020 instrument, and the adsorption tube was kept at a constant temperature of 273 K, 296 K and 313 K by using a Julabo water bath. N_2_ sorption isotherms were recorded by using a Micromeritics ASAP 2460 instrument at 77 K under liquid N_2_ bath. Kinetic and equilibrium adsorption measurements were carried out using an Intelligent Gravimetric Analyzer (IGA001, Hiden, UK) under diverse test conditions.

### Single-crystal X-ray diffraction

SCXRD data were collected at 170 K for Al-PyDC-hydrated, and at 200 K for C_2_H_2_@Al-PyDC, C_2_H_6_@Al-PyDC and C_2_H_4_@Al-PyDC on an Agilent Supernova CCD diffractometer equipped with graphite-monochromatic enhanced Cu-Kα radiation (*λ* = 1.54184 Å). A single crystal of solvent-exchanged Al-PyDC was selected and placed into a capillary glass tube with inner diameter of 0.1 mm. This crystal was activated at 393 K for 4 h, and the capillary glass tube was filled by pure C_2_H_2_, C_2_H_6_ or C_2_H_4_ gas up to 1 bar and then sealed to obtain C_2_H_2_-loaded, C_2_H_6_-loaded or C_2_H_4_-loaded Al-PyDC crystal. The datasets were corrected by empirical absorption correction using spherical harmonics, implemented in the SCALE3 ABSPACK scaling algorithm. The structure was solved by direct methods and refined by full matrix least-squares methods with the SHELX-97 program package. During crystal structure analysis for C_2_H_6_@Al-PyDC, we found that guest C_2_H_6_ molecules exhibited highly positional disorder within the channels. By atomic identification and refinement, we determined that the asymmetric unit of each C_2_H_6_ molecule within the channel includes four carbon atoms (C5, C6, C8, C9) and eight hydrogen atoms (H5A, H5C, H6A, H6C, H8A, H8C, H9A, H9C). Among them, four carbon atoms and four hydrogen atoms (H5A, H6A, H8A, H9A) are located on a mirror plane (−*x*, *y*, *z*), while two carbon atoms (C6 and C8) are located on a mirror plane (*x*, 1/2−*y*, *z*) and a 2-fold rotation axis (1/2−*x*, *y*, 1/2−*z*). The asymmetric unit of C_2_H_6_ is generated by these symmetry operations, resulting in four completely disordered C_2_H_6_ molecules at each site, with an occupancy of 25%. All the crystal data are summarized in Supplementary Table 4, and ORTEP style illustrations of all structures are provided in Supplementary Figs. 31–34.

### Breakthrough experiments

The breakthrough curves were obtained from a dynamic gas breakthrough apparatus equipped with stainless steel column (*Φ* 4 × 120 mm). The weight of sample packed in the column was 0.34 g. The mixed gas flows of (1) C_2_H_2_/C_2_H_4_ (1/99, v/v), (2) C_2_H_6_/C_2_H_4_ (50/50), (3) C_2_H_2_/C_2_H_6_/C_2_H_4_ (1/49.5/49.5) or (4) C_2_H_2_/C_2_H_6_/C_2_H_4_ (1/9/90) were introduced into breakthrough apparatus with the rate of 1.25, 5 and 10 mL min^−1^ at 296 K and 1 bar, respectively. The outlet gas was monitored using a gas chromatography (GC-2014C, SHIMADZU) equipped with thermal conductivity detector (TCD, detection limit 0.1 ppm). The concentration of the outlet gas was calibrated by detecting the standard gas mixture. After each breakthrough experiment, the sample can be regenerated under a purging He gas with a flow of 10 mL min^−1^ at 373 K for 1 h.

### Cycling experiments

The cycling experiments for single-component C_2_H_2_ or C_2_H_6_ were performed on a Micromeritics ASAP 2020 surface area analyzer. The activated sample was exposed to C_2_H_2_ or C_2_H_6_ gas at 1 bar and 296 K until saturation, followed by desorption under vacuum at room temperature to 0.001 bar. The cycling experiments for C_2_H_6_ with 1000 ppm H_2_S were measured by using DSC/TGA Discovery SDT 650. After being fully activated at 393 K under N_2_ flow for 2 h, the adsorbent was exposed to C_2_H_6_ gas containing 1000 ppm H_2_S (100 mL min^−1^) at 296 K for 60 min, followed by regeneration at 373 K under N_2_ flow (200 mL min^−1^) for 30 min. Cycling breakthrough experiments for C_2_H_2_/C_2_H_6_/C_2_H_4_ mixture were performed using a dynamic gas breakthrough apparatus equipped with stainless steel column. The weight of sample packed in the column was 0.418 g. The mixed gas flows of (1) C_2_H_2_/C_2_H_6_/C_2_H_4_ (1/49.5/49.5), (2) C_2_H_2_/C_2_H_6_/C_2_H_4_ (1/9/90) or (3) C_2_H_2_/C_2_H_6_/C_2_H_4_ (1/9/90) with 1000 ppm H_2_S were introduced into breakthrough apparatus with the rate of 1.25 mL min^−1^ at 296 K and 1 bar. After each breakthrough experiment, the sample can be fully regenerated under a purging He gas with a flow of 10 mL min^−1^ at 373 K for 30 min.

### Supplementary information

Supplementary Information File

Peer Review File

## Data Availability

Crystallographic data for the structures in reported this article have been deposited at the Cambridge Crystallographic Data Centre, under deposition numbers CCDC 2242152−2242155. Copies of the data can be obtained free of charge via https://www.ccdc.cam.ac.uk/structures/. All the other relevant data that support the findings of this study are available within the article and its Supplementary Information, or from the corresponding author upon request.
