# Supplementary material for: Incorporation of multiple supramolecular binding sites into a robust MOF for benchmark one-step ethylene purification
Source: Nat Commun. 2023 Oct 2;14:6146. doi: 10.1038/s41467-023-41692-x (PMC10545795; doi:10.1038/s41467-023-41692-x)
Supplement: Supplementary file 1 — Supplementary Information File [file 41467_2023_41692_MOESM1_ESM.pdf]

Supplementary Information

Incorporation of multiple supramolecular binding sites into a robust MOF for benchmark one-step ethylene purification

Enyu Wu[1+], Xiao-Wen Gu[1+], Di Liu[1], Xu Zhang[2], Hui Wu[3], Wei Zhou[3], Guodong Qian[1] and Bin Li[1]*

[1] State Key Laboratory of Silicon and Advanced Semiconductor Materials, School of Materials Science and Engineering, Zhejiang University, Hangzhou 310027, China.

[2] School of Chemistry and Chemical Engineering, Huaiyin Normal University, Huaian 223300, China.

[3] NIST Center for Neutron Research, National Institute of Standards and Technology, Gaithersburg, MD 20899-6102, USA.

[+] These authors contributed equally to this work.

*E-mail: bin.li@zju.edu.cn

Table of Contents

**Supplementary Notes**	S3
**Supplementary Discussion**	S4
**Supplementary Tables**	S7
**Supplementary Figures**	S14
**Supplementary References**	S31

Supplementary Notes

IAST calculations

The IAST was used to predict the binary mixture adsorption from the experimental pure gas isotherms. To perform the integrations required by IAST, single-component isotherms should be fitted by the correct model. The experimental pure component isotherm data for C_2H_2, C_2H_6 and C_2H_4 in Al-PyDC measured at 296 K were fitted with single-site Langmuir-Freundlich equation.

$$q = q_{\text{sat}} \frac{bp^{v}}{1+bp^{v}} \quad (1)$$

with T-dependent parameters b

$$b = b_0 \exp\left(\frac{E}{RT}\right) \quad (2)$$

Here, p is the pressure of the bulk gas in equilibrium with the adsorbed phase (kPa), q is the amount adsorbed per mass of adsorbent (mmol g^{-1}), q_{sat} is the saturation capacities of site 1 (mmol g^{-1}), b is the affinity coefficients of site 1 (kPa^{-1}) and v represents the deviations from an ideal homogeneous surface. The fitted parameter values are presented in Supplementary Table 2.

The fitted parameters were then used to predict multi-component adsorption with IAST. The selectivity of preferential adsorption of component 1 over component 2 in a mixture containing 1 and 2, can be formally defined as:

$$S_{\text{ads}} = \frac{q_1/q_2}{p_1/p_2} \quad (3)$$

In equation (3), q_1 and q_2 are the molar loadings of the adsorbed phase in equilibrium with the bulk gas phase with partial pressures p_1 and p_2. IAST calculations of the adsorption selectivity for 1/99 (v/v) C_2H_2/C_2H_4 and 50/50 (v/v) C_2H_6/C_2H_4 mixtures in Al-PyDC at 296 K are shown in Fig. 3c.

Isosteric heat of adsorption

A virial-type expression of comprising the temperature-independent parameters a_i and b_j was employed to calculate the enthalpies of adsorption for C_2H_2, C_2H_4 and C_2H_6 on Al-PyDC. In each case, the data were fitted with equation:

$$\ln P = \ln N + 1/T \sum_{i=0}^{m} a_i N_i + \sum_{j=0}^{n} b_j N_j \quad (4)$$

Here, P is the pressure expressed in Pa, N is the amount absorbed in mmol g^{-1}, T is the temperature in K, a_i and b_j are virial coefficients, and m, n represent the number of coefficients required to adequately describe the isotherms (m and n were gradually increased till the contribution of extra added a and b coefficients was deemed to be statistically insignificant towards the overall fit. The average value of the squared deviations from the experimental values was minimized). The values of the virial coefficients a_0 to a_m were then used to calculate the isosteric heat of adsorption utilizing the following expression:

$$Q_{st} = -R\sum_{i=0}^{m} a_i N_i \qquad (5)$$

Q_{st} is the coverage-dependent isosteric heat of adsorption and R is the universal gas constant. The heat enthalpies of C_2H_2, C_2H_6 and C_2H_4 sorption for Al-PyDC in this manuscript are determined by using the sorption data measured in the pressure range from 0 to 1 bar (at 273 K, 296 K and 313 K).

Supplementary Discussion

C_2 adsorption at different temperatures

Since the C_2H_2 and C_2H_6 binding affinity are stronger than C_2H_4 (Supplementary Fig. 12), it is normal that the C_2H_2 and C_2H_6 uptakes of Al-PyDC are higher than that of C_2H_4 at different temperatures. As shown in Fig. 3b and Supplementary Fig. 5, Al-PyDC indeed exhibits more obviously preferential adsorption of C_2H_2 (8.24 and 5.80 mmol g^{-1} at 1 bar) and C_2H_6 (4.20 and 3.02 mmol g^{-1}) over C_2H_4 (3.44 and 2.65 mmol g^{-1}) at 296 K and 313 K. However, at 273 K, the C_2H_4 and C_2H_6 uptakes (5.0 and 5.13 mmol g^{-1}) are quite similar at 1 bar, accompanied with the preferential adsorption of C_2H_6 over C_2H_4 at low-pressure regions. To explain this abnormal behavior between C_2H_6 and C_2H_4 at 273 K, our gas-loaded crystal structures experimentally revealed that the main binding sites for C_2H_6 and C_2H_4 are the same but with different binding strength (Figs. 4e and 4f). The full occupancy of these main binding sites corresponds to the saturated adsorption amount of 5.08 mmol g^{-1}. At 273 K, the quite similar C_2H_6 and C_2H_4 uptakes (5.0 and 5.13 mmol g^{-1}) are consistent well with the saturated amount obtained from gas-loaded crystal structures. This indicates that the main binding sites are fully occupied for both C_2H_4 and C_2H_6 at 273 K. With the adsorption temperature increased to 296

K or 313 K, the adsorption occupancy of the main binding sites would be reduced for all C_2 gases; however, the stronger binding affinity of C_2H_6 results in a higher adsorption occupancy of the main binding sites than C_2H_4 (82% vs 68% at 296 K, and 59% vs 52% at 313 K). The same situation was also observed for C_2H_2 adsorption, in which C_2H_2 uptake at 273 K (10.34 mmol g^{-1}) matches with the full occupation of the main binding sites (10.1 mmol g^{-1}) obtained by C_2H_2-loaded crystal structure. With the temperature increased to 296 and 313 K, the adsorption occupancy of the main binding sites was decreased to be 82% at 296 K and 58% at 313 K. Such stronger binding affinity to result in higher adsorption occupancy of the main binding sites was also exemplified in other materials for reversed C_2H_4/C_2H_6 separation.[1]

Therefore, the similar C_2H_6 and C_2H_4 uptakes at 273 K are mainly dominated by the full occupancy of the main binding sites for both gases. With the adsorption temperature increased to 296 K and 313 K, the stronger binding affinity of C_2H_6 enables a higher adsorption occupancy of the main binding sites than C_2H_4, thus resulting in the more obviously preferential adsorption of C_2H_6 over C_2H_4 at 296 K and 313 K than at 273 K.

Notation

q	Component molar loading of species i, mol kg^{-1}
q_{sat}	Saturation loading, mol kg^{-1}
b	Langmuir-Freundlich constant, kPa^{-v}
T	Absolute temperature, K
a_i	Virial coefficients, dimensionless
b_j	Virial coefficients, dimensionless
Q_{st}	Isosteric heat of adsorption, kJ mol^{-1}

Greek letters

v	Freundlich exponent, dimensionless

Supplementary Tables

Supplementary Table 1. Physical properties of C_2H_2, C_2H_4, and C_2H_6.

Molecular formula	Molecular dimension (Å)			Kinetic diameter (Å)	Polari × 10^{-25} (cm^{-3})	Boiling point (K)
	X	*Y*	*Z*			
C_2H_2	3.32	3.34	5.70	3.33	33.3−33.9	188.4
C_2H_4	3.28	4.18	4.84	4.16	42.5	169.4
C_2H_6	3.81	4.08	4.82	4.44	44.3−44.7	184.6

Supplementary Table 2. Single-site Langmuir-Freundlich parameter fits for C_2H_2, C_2H_4 and C_2H_6 in Al-PyDC. The fits are based on experimental isotherm data at 296 K.

	q_{sat} mol kg^{-1}	b kPa^{-v}	v dimensionless
C_2H_2	14.09674	6.20419	6.37767
C_2H_4	0.023	0.01372	0.0255
C_2H_6	0.87198	1.00448	0.92349

Supplementary Table 3. Summary of adsorption and separation metrics of C_2H_2/C_2H_6-selective materials reported in the literatures at 1 bar and room temperature.

MOFs	C_2H_2 uptake[a] (mmol g^{-1})	C_2H_6 uptake[a] (mmol g^{-1})	C_2H_2/C_2H_4 selectivity[b]	C_2H_6/C_2H_4 selectivity[c]	Ref.
**Al-PyDC**	**8.24**	**4.20**	**4.3**	**1.9**	**This work**
Zn(ad)(int)	2.90	2.32	1.61	2.4	2
$CuTiF_6$-TPPY	3.62	2.82	5.03	2.12[e]	3
Zn(BDC)(H_2BPZ)	4.46	3.60	1.6[d]	2.2	4
UiO-67-$(NH_2)_2$	5.90	5.32	2.1	1.7	5
NPU-1	5.09	4.50	1.4[d]	1.32	6
NPU-2	4.02	4.43	1.25[d]	1.52	6
TJT-100	4.46	3.75	1.8	1.2[f]	7
Azole-Th-1	3.62	4.47	1.0	1.46	8
NUM-9	2.36	2.47	1.47	1.62	9
UPC-612	3.01	3.57	1.07[e]	1.41	10
UPC-613	2.83	2.54	1.4[e]	1.47	10
MOF-525	2.64	2.70	1.44[e]	1.25	10
MOF-525(Co)	2.62	2.21	1.87[e]	1.1	10
Ag-PCM-102	4.59	3.69	1.5	–	11

[a] At 1 bar and room temperature.

[b] IAST selectivity for 1/99 C_2H_2/C_2H_4 gas mixture.

[c] IAST selectivity for 50/50 C_2H_6/C_2H_4 gas mixture.

[d] Selectivity is for a 50/50 mixture.

[e] Selectivity is for a 10/90 mixture.

[f] Selectivity is for a 1/99 mixture.

Supplementary Table 4. Crystallographic data and structure refinement results of C_2H_2-loaded Al-PyDC, C_2H_4-loaded Al-PyDC, C_2H_6-loaded Al-PyDC and Al-PyDC-hydrated.

Unit cell parameters	C_2H_2@Al-PyDC	C_2H_4@Al-PyDC	C_2H_6@Al-PyDC	Al-PyDC-hydrated
Formula	$C_{20}H_{16}Al_2N_2O_{10}$	$C_{16}H_{16}Al_2N_2O_{10}$	$C_{16}H_{20}Al_2N_2O_{10}$	$C_{24}H_{49}Al_4N_4O_{38}$
Formula weight	498.31	450.27	454.30	1109.59
Temperature/K	200(2)	200(2)	200(2)	170(2)
Crystal system	Tetragonal	Tetragonal	Tetragonal	Tetragonal
Space group	$I4_1$/amd	$I4_1$/amd	$I4_1$/amd	$I4_1$md
a, b (Å)	21.2209(11)	21.198(3)	21.2349(11)	21.1895(3)
c (Å)	10.6352(15)	10.675(4)	10.6874(11)	10.6283(3)
α (°)	90	90	90	90
β (°)	90	90	90	90
γ (°)	90	90	90	90
V (Å^3)	4789.3(8)	4797(2)	4819.2(7)	4772.05(19)
Z	8	8	8	4
D_{calcd} (g cm^{-3})	1.382	1.247	1.252	1.544
μ (mm^{-1})	1.609	1.547	1.540	1.954
F(000)	2048	1856	1888	2308
GOF	1.115	1.044	1.066	1.060
R_{int}	0.2574	0.3459	0.3499	0.1597
R_1, wR_2 [I>=2σ (I)]	0.1308, 0.3769	0.1644, 0.4952	0.1322, 0.3891	0.0545, 0.1462
R_1, wR_2 [all data]	0.1944, 1.106	0.2788, 1.037	0.1791, 2.791	0.0721, 1.059
Largest diff. peak and hole (e Å^{-3})	0.578 and -0.656	1.077 and -0.423	1.012 and -0.607	0.939 and -0.702
CCDC number	2242152	2242153	2242154	2242155

Supplementary Table 5. The interactions between the C_2H_2 molecule and host framework in Al-PyDC, determined by C_2H_2-loaded single-crystal X-ray diffraction studies.

			D···A (Å)[a]	H···A (Å)[b]
C_2H_2	Site I	C−H···O	3.24	2.47
			3.24	2.47
			3.44	3.13
			3.44	3.13
			3.36	3.30
			3.36	3.30
		O−H···C	2.90	1.98
			2.90	1.98
		$C{-}H_{pyrrole}\cdots C_{C2H2}$	3.26	2.40
			3.26	2.40
	Site II	C−H···O	3.36	2.41
			3.73	3.12
			3.91	3.35
		N−H···C	3.01	2.32
			3.30	2.88
		C−H···π	4.10	3.18
	Cluster	$C{-}H_{C2H2}\cdots C_{C2H2}$	4.09	3.78
			4.50	3.98
			4.50	4.40
			4.91	4.40

[a] The distance between the donor atom and the acceptor atom. [b] The distance between the hydrogen atom and the acceptor atom.

Supplementary Table 6. The interactions between the C_2H_6 molecule and host framework in Al-PyDC, determined by C_2H_6-loaded single-crystal X-ray diffraction studies..

		D···A (Å)[a]	H···A (Å)[b]
C_2H_6	C−H···O	4.32	3.54
		4.32	3.54
		4.76	4.08
		4.76	4.08
		4.43	4.36
		4.43	4.36
		5.12	4.37
		5.12	4.37
		4.72	4.42
		4.72	4.42
		5.41	4.51
		5.41	4.51
		5.60	4.65
		5.60	4.65
	C−H···N	4.16	3.80
		4.16	3.80
		4.74	4.18
	C−H···π	4.07	3.33
		4.07	3.33
		4.35	3.71
		4.35	3.71

[a] The distance between the donor atom and the acceptor atom. [b] The distance between the hydrogen atom and the acceptor atom.

Supplementary Table 7. The interactions between the C_2H_4 molecule and host framework in Al-PyDC, determined by C_2H_4-loaded single-crystal X-ray diffraction studies..

		D···A (Å)[a]	H···A (Å)[b]
C_2H_4	C−H···O	4.37	3.78
		4.37	3.78
		4.56	3.83
		4.56	3.83
		4.17	3.91
		4.17	3.91
		4.53	4.08
		4.53	4.08
	C−H···N	4.04	3.21
		4.17	3.42
	C−H···π	4.54	4.26
		4.54	4.26

[a] The distance between the donor atom and the acceptor atom. [b] The distance between the hydrogen atom and the acceptor atom.

Supplementary Table 8. Comparison of stability and synthetic conditions of Al-PyDC with reported C_2H_2/C_2H_6-selective MOFs and other representative stable MOFs.

	MOFs	Stability			Synthetic conditions			Ref.
		Water	pH=1	pH=12	Temp (°C)	Time (h)	Solvent	
C_2H_2/C_2H_6-selective MOFs	**Al-PyDC**	**√**	**√**	**√**	**85**	**9**	**H_2O**	**This work**
	Zn(ad)(int)	√	–[a]	–	120	72	DMF	3
	Zn(BDC)(H_2BPZ)	–	–	–	120	576	DMF	4
	UiO-67-(NH_2)$_2$	√	–	√	120	48	DMF	5
	NPU-1	–	–	–	100	240	DMA	6
	TJT-100	√	–	√	150	72	DMF/H_2O	7
	Azole-Th-1	√	√	√	110	72	DMF	8
	NUM-9	–	–	–	100	72	DMA/EtOH/H_2O	9
	UPC-612	√	√	√	120	48	DMF	10
	MOF-525	–	–	–	120	48	DMF	10
Stable MOFs	MIL-53(Al)	√	–	√	220	72	H_2O	12
	UiO-66	√	√	√	120	24	DMF	13
	ZIF-8	√	–	√	140	24	DMF	14
	MIL-101(Cr)	√	√	√	220	8	H_2O	15
	PCN-250	√	√	√	140	12	DMF	16
	BUT-12	√	√	–	120	48	DMF	17

[a] Stability in the corresponding conditions was not reported in literatures.

Supplementary Figures

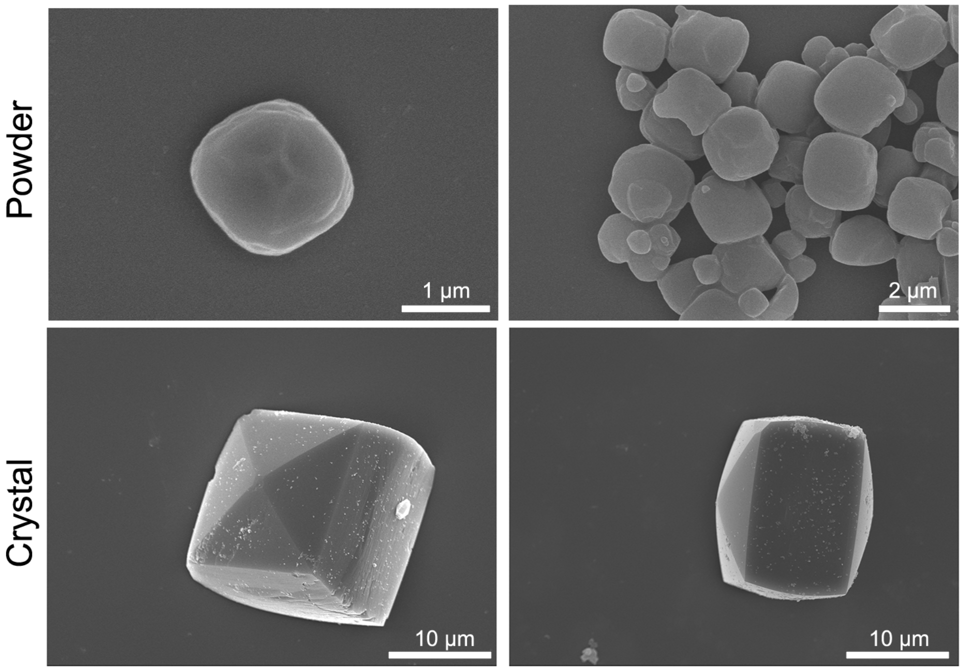

Supplementary Fig. 1 SEM images. SEM images of Al-PyDC powder obtained from conventional reflux method, and single crystal obtained from the crystal synthesis method.

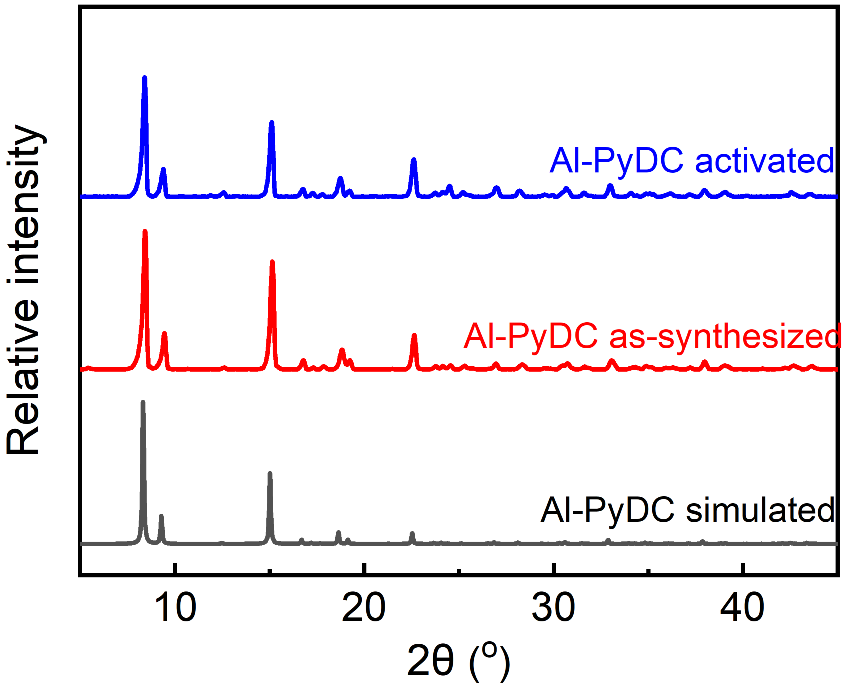

Supplementary Fig. 2 PXRD patterns. The calculated PXRD pattern from the model structure of Al-PyDC and PXRD patterns of as-synthesized Al-PyDC, activated Al-PyDC.

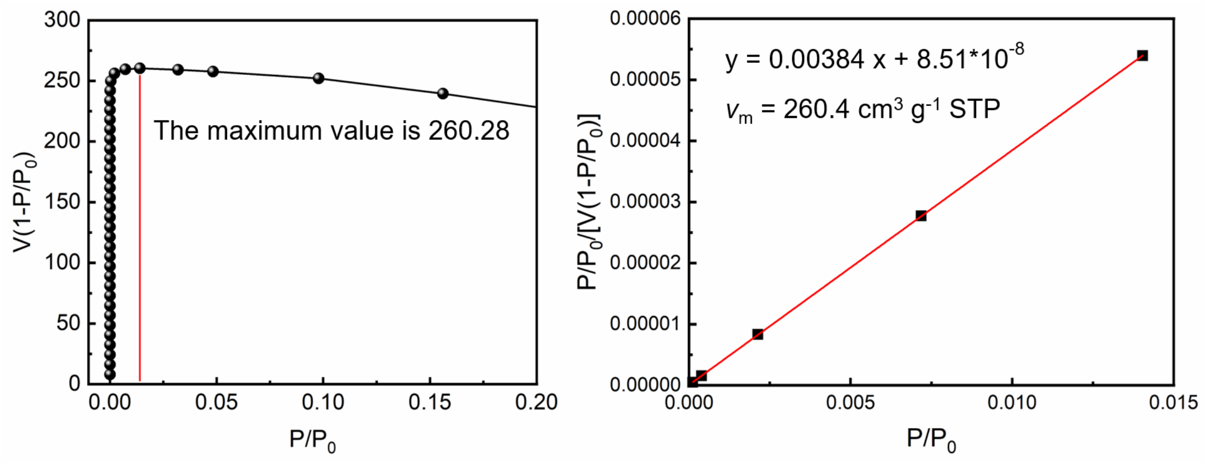

Supplementary Fig. 3 Fit curves. Al-PyDC BET specific surface area fitting.

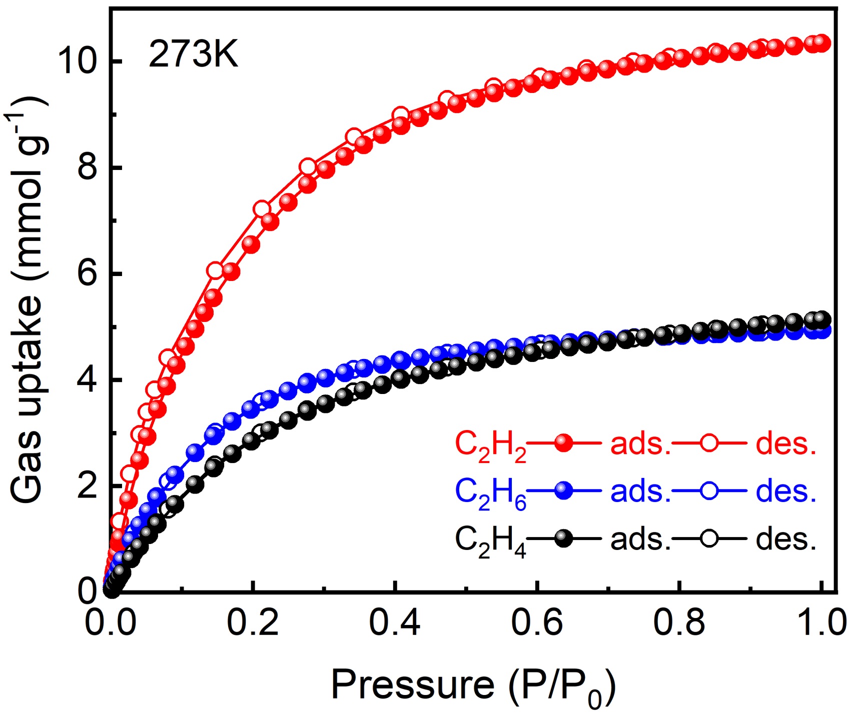

Supplementary Fig. 4 C_2 adsorption. Adsorption isotherms of C_2H_2 (red), C_2H_6 (blue) and C_2H_4 (black) for Al-PyDC at 273 K.

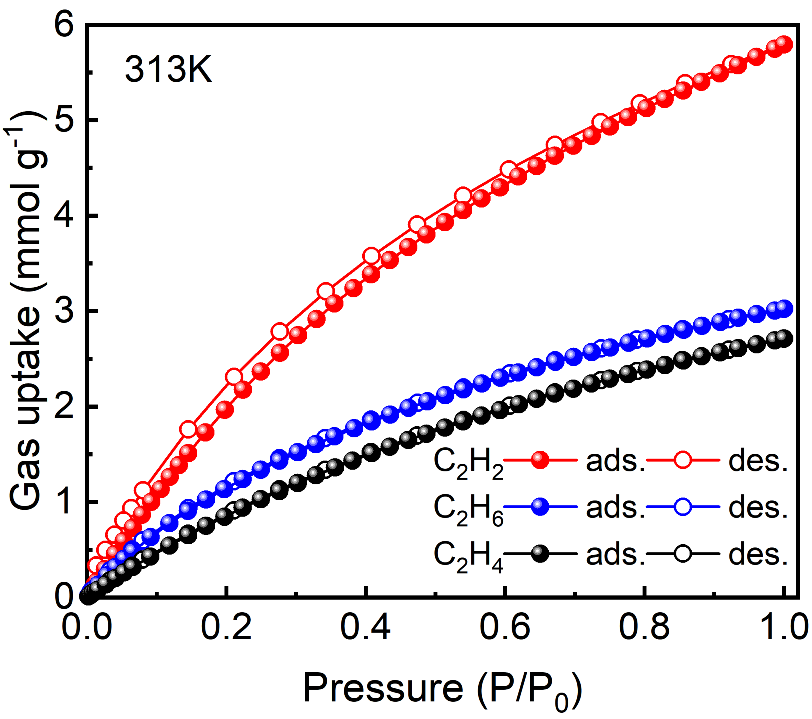

Supplementary Fig. 5 C_2 adsorption. Adsorption isotherms of C_2H_2 (red), C_2H_6 (blue) and C_2H_4 (black) for Al-PyDC at **a** 296 K and **b** 313 K.

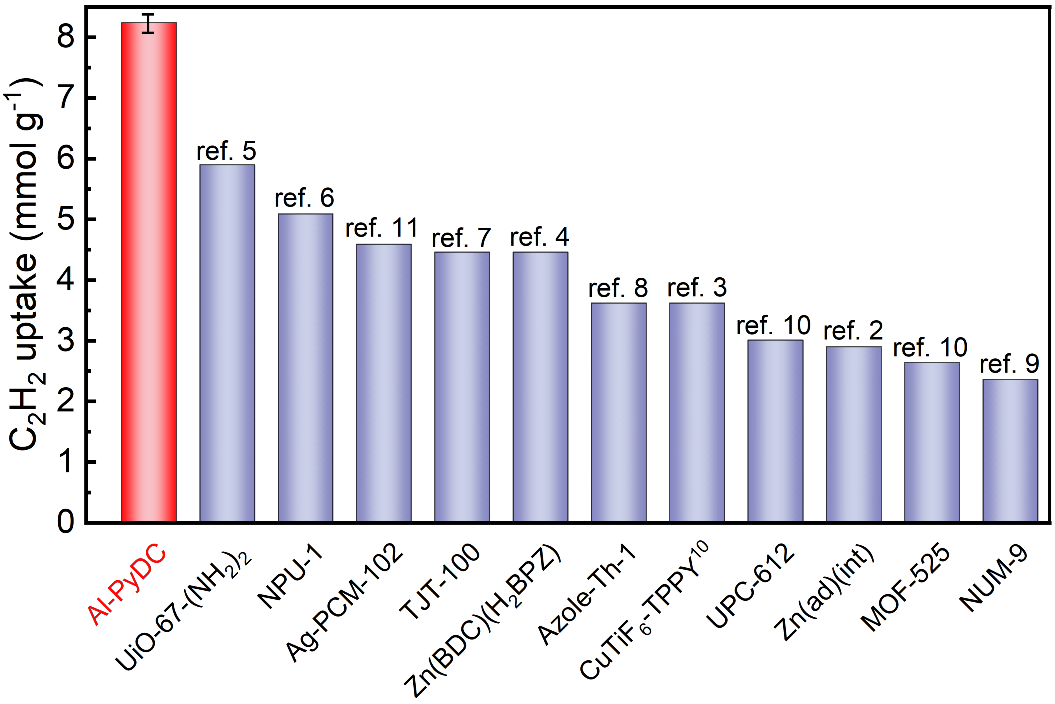

Supplementary Fig. 6 Comparison of adsorption capacity. Comparison of C_2H_2 uptake for Al-PyDC (the averaged value was obtained from five independent tests, and the error bar is the standard deviation) with the indicated benchmark MOFs at ambient conditions.

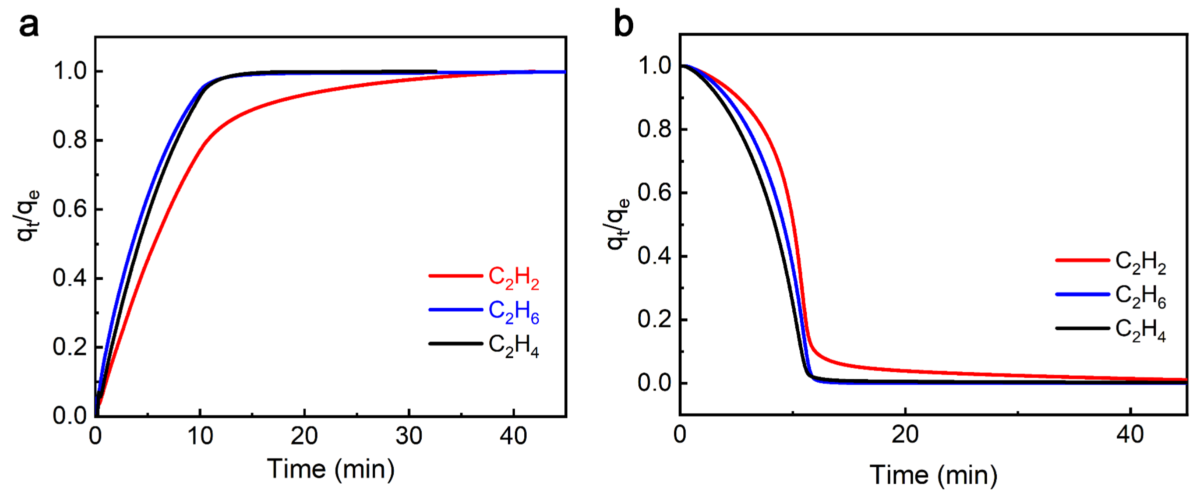

Supplementary Fig. 7 Kinetics profiles. **a** Adsorption kinetics profiles of C_2H_2 (red), C_2H_6 (blue) and C_2H_4 (black) for activated Al-PyDC at 296 K and 1 bar. **b** Desorption kinetics profiles of C_2H_2 (red), C_2H_6 (blue) and C_2H_4 (black) for saturated Al-PyDC at 296 K and 10^{-5} bar. q_t/q_e represents the gravimetric uptake/total equilibrium gravimetric uptake (at 1 bar), indicating the gas sorption rate of materials.

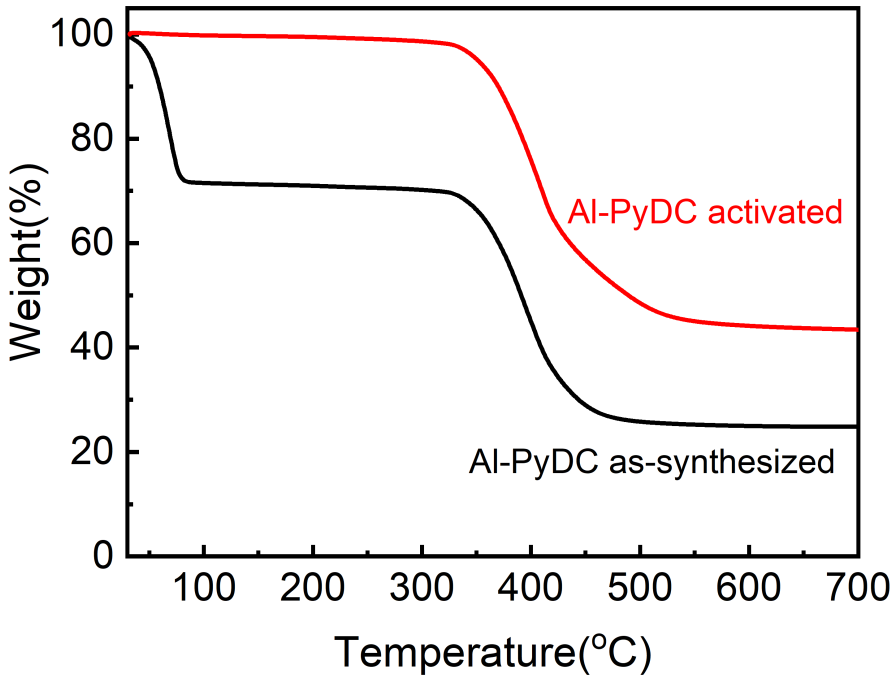

Supplementary Fig. 8 TGA. TGA analysis curve of as-synthesized (black) and activated (red) Al-PyDC. The data were collected under 5 K min^{-1} of heating rate and 30 mL min^{-1} N_2 flow.

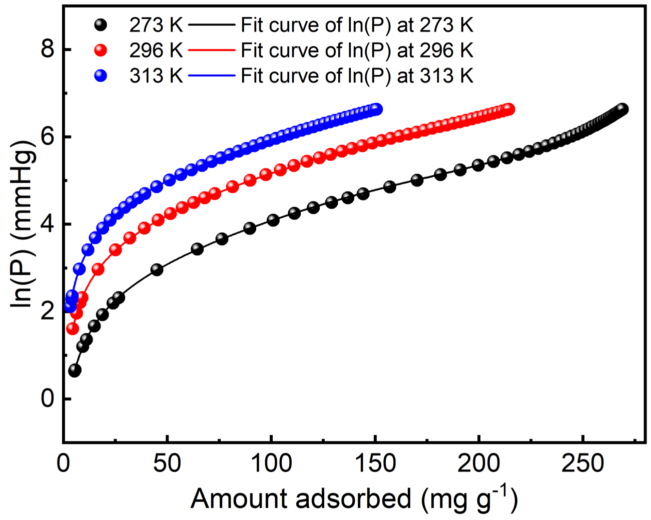

Equation	y=ln(x)+1/k*(a0+a1*x+a2*x^2+a3*x^3 +a4*x^4+a5*x^5)+(b0+b1*x+b2*x^2)	
Adj. R-suqre	0.99999	
	Value	Standard Erro
a0*	-4273.31733	10.4357
a1*	4.99444	0.24812
a2*	-0.02379	0.00227
a3*	1.19E-04	2.15E-05
a4*	-8.38E-07	8.87E-08
a5*	1.83E-09	1.30E-10

Supplementary Fig. 9 Fit curves. Virial fitting of the C_2H_2 adsorption isotherms for Al-PyDC.

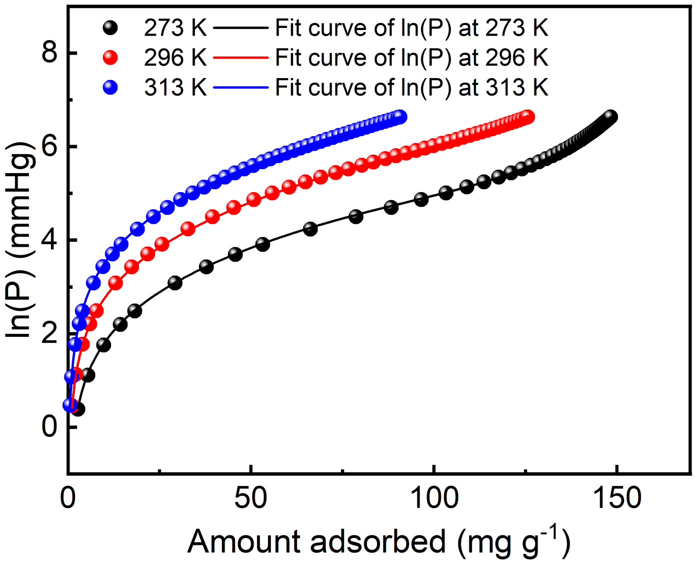

Equation	y=ln(x)+1/k*(a0+a1*x+a2*x^2+a3*x^3 +a4*x^4+a5*x^5)+(b0+b1*x+b2*x^2)	
Adj. R-suqre	0.99999	
	Value	Standard Erro
a0*	-3619.84812	23.43929
a1*	-0.55603	0.94057
a2*	-0.05059	0.015
a3*	5.90E-04	2.40E-04
a4*	-7.62E-06	1.76E-06
a5*	3.33E-08	4.65E-09

Supplementary Fig. 10 Fit curves. Virial fitting of the C_2H_6 adsorption isotherms for Al-PyDC.

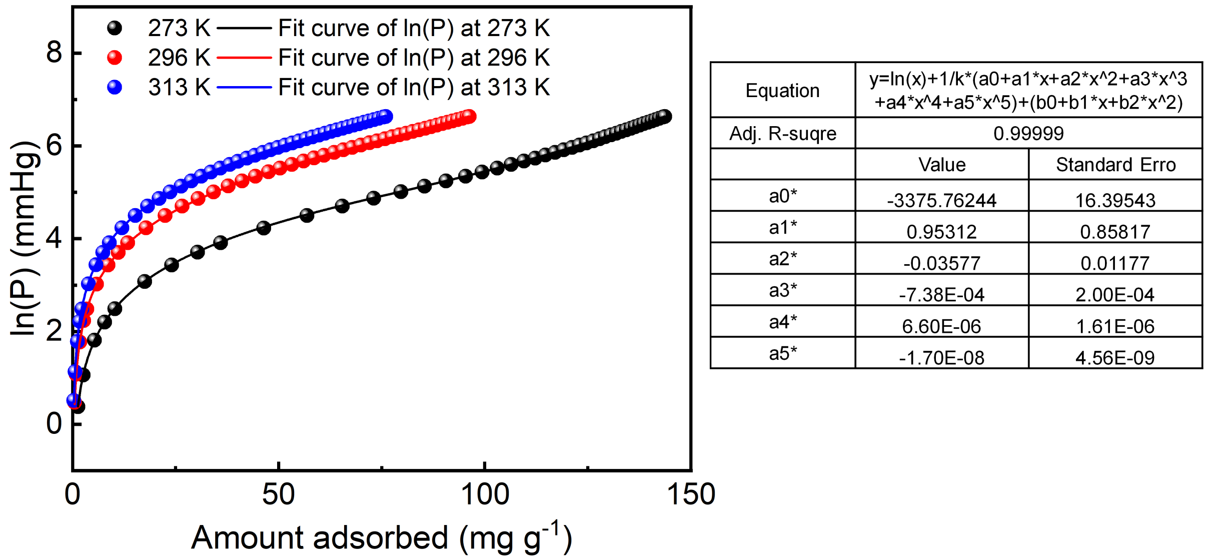

Equation	y=ln(x)+1/k*(a0+a1*x+a2*x^2+a3*x^3 +a4*x^4+a5*x^5)+(b0+b1*x+b2*x^2)	
Adj. R-suqre	0.99999	
	Value	Standard Erro
a0*	-3375.76244	16.39543
a1*	0.95312	0.85817
a2*	-0.03577	0.01177
a3*	-7.38E-04	2.00E-04
a4*	6.60E-06	1.61E-06
a5*	-1.70E-08	4.56E-09

Supplementary Fig. 11 Fit curves. Virial fitting of the C_2H_4 adsorption isotherms for Al-PyDC.

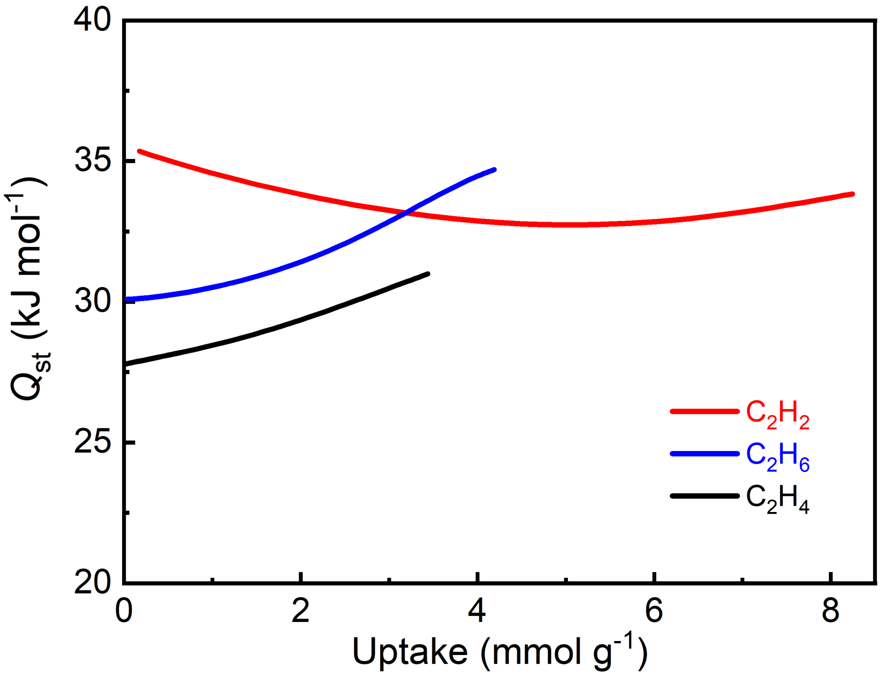

Supplementary Fig. 12 Q_{st}. The Q_{st} of C_2H_2, C_2H_6 and C_2H_4 for Al-PyDC.

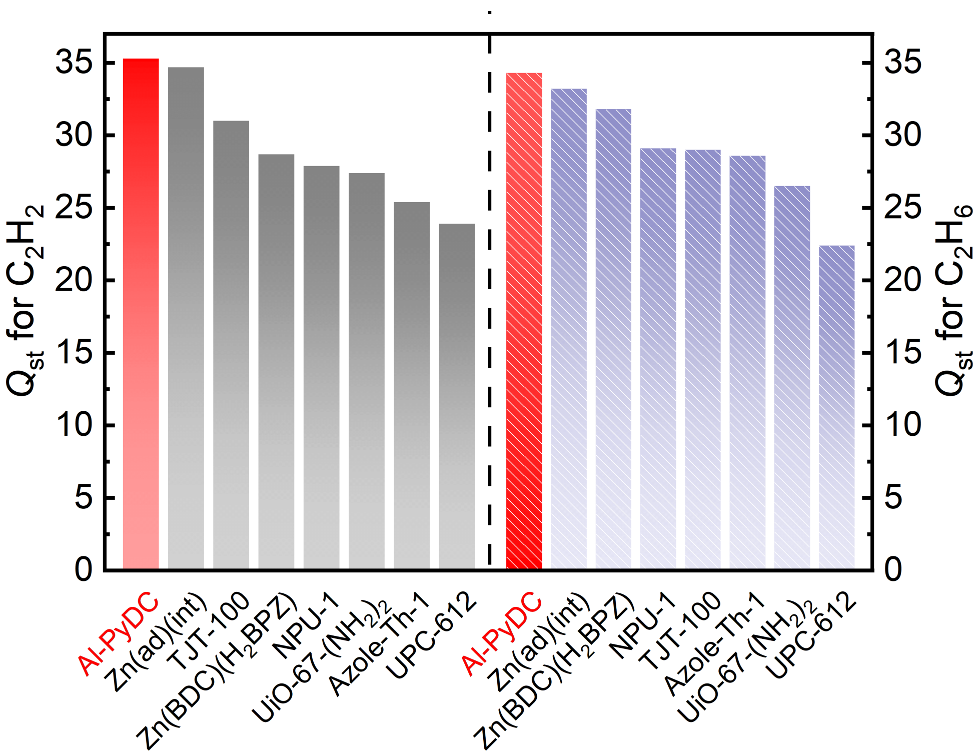

Supplementary Fig. 13 Comparison of maximum Q_{st}. The comparison of maximum Q_{st} for C_2H_2 and C_2H_6.

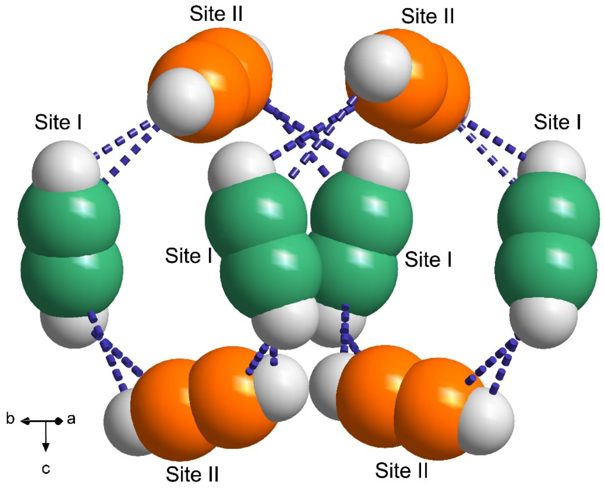

Supplementary Fig. 14 Binding sites. The C_2H_2 cluster in C_2H_2-loaded Al-PyDC determined by SCXRD analysis. The Al-PyDC framework is omitted for clarity.

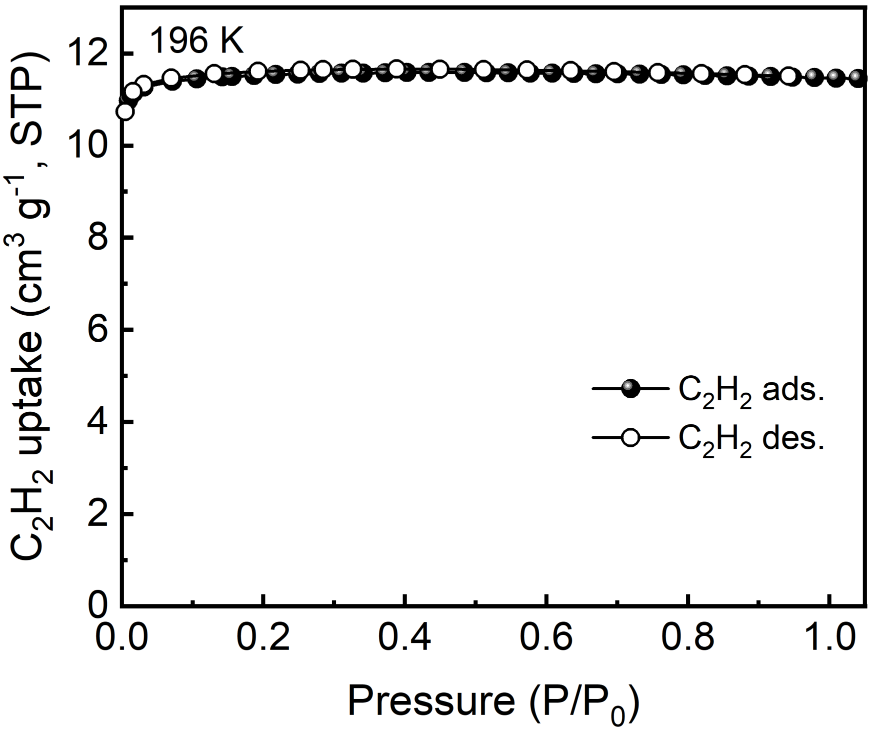

Supplementary Fig. 15 C_2H_2 sorption. C_2H_2 sorption isotherms of Al-PyDC at 196 K.

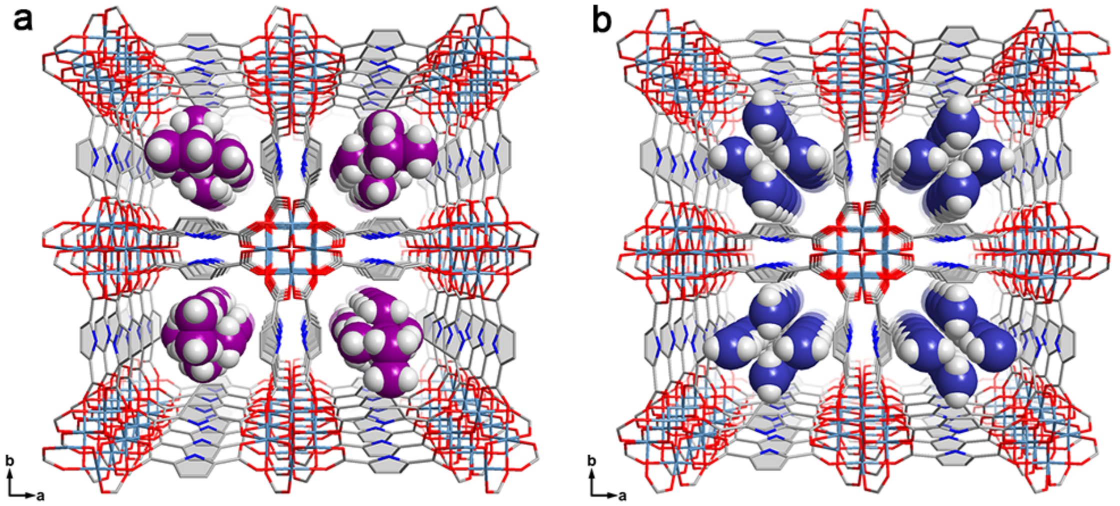

Supplementary Fig. 16 Binding sites. The SCXRD structures of **a** C_2H_6-loaded and **b** C_2H_4-loaded Al-PyDC, viewed along the *c* axis.

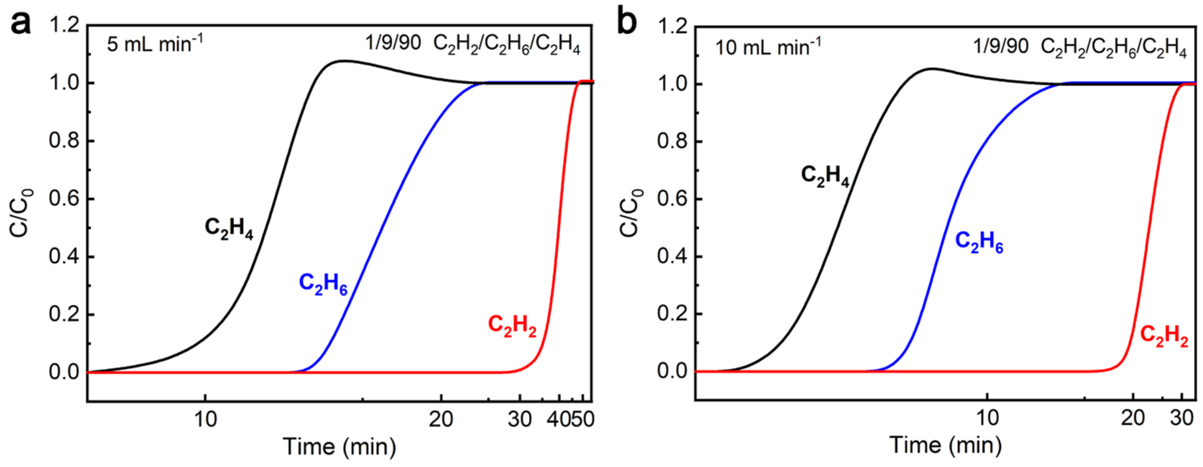

Supplementary Fig. 17 Breakthrough curves. Experimental column breakthrough curves for $C_2H_2/C_2H_6/C_2H_4$ (1/9/90) mixture with a total flow of **a** 5 mL min^{-1} and **b** 10 mL min^{-1} in an absorber bed packed with Al-PyDC at 296 K and 1 bar.

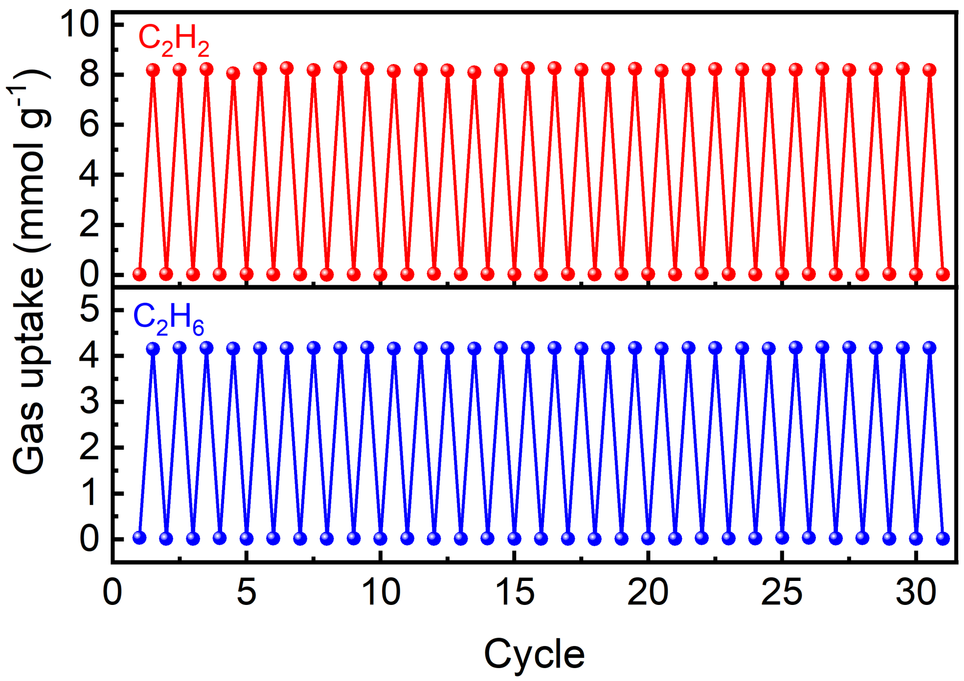

Supplementary Fig. 18 Recyclability. The repeated adsorption-desorption cycles of Al-PyDC for C_2H_2 and C_2H_6 sorption at 296 K between the pressure of 1 bar and 0.001 bar.

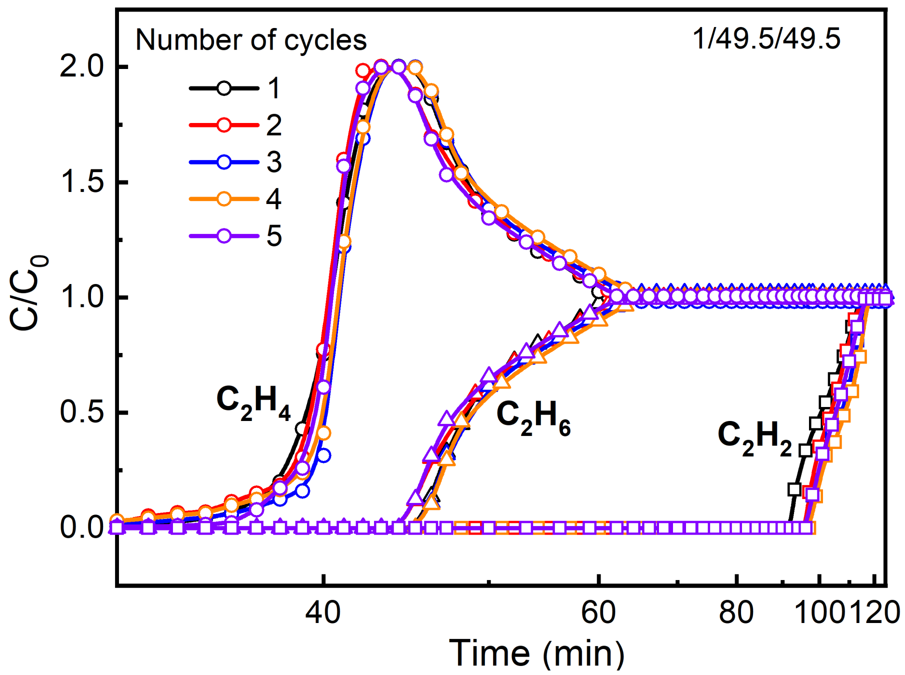

Supplementary Fig. 19 Recyclability. The five repeated separation cycles of breakthrough experiments on Al-PyDC for the 1/49.5/49.5 $C_2H_2/C_2H_6/C_2H_4$ mixture.

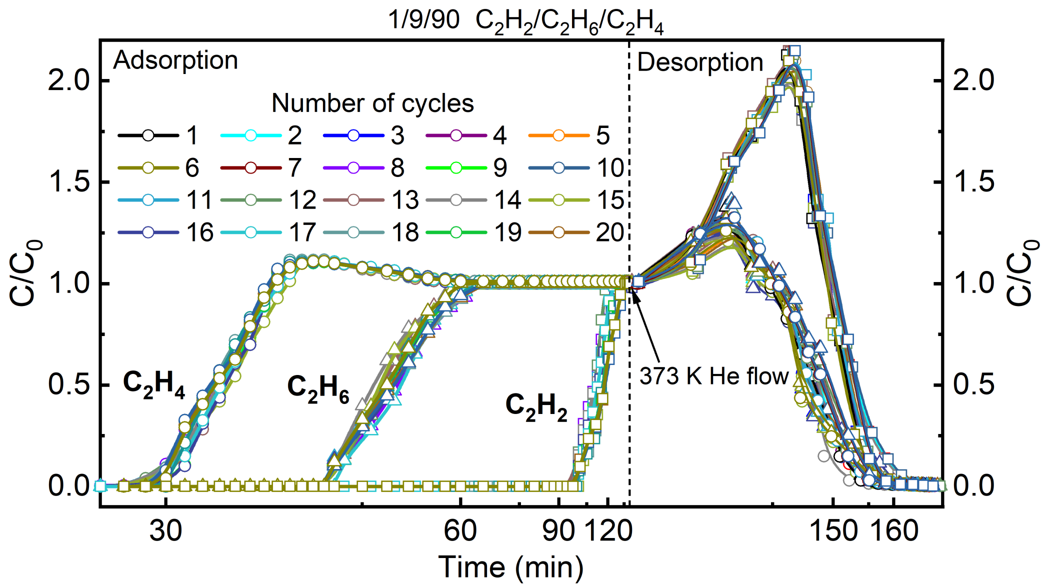

Supplementary Fig. 20 Recyclability. The twenty repeated adsorption-desorption cycles of breakthrough experiments on Al-PyDC for the 1/9/90 $C_2H_2/C_2H_6/C_2H_4$ mixture.

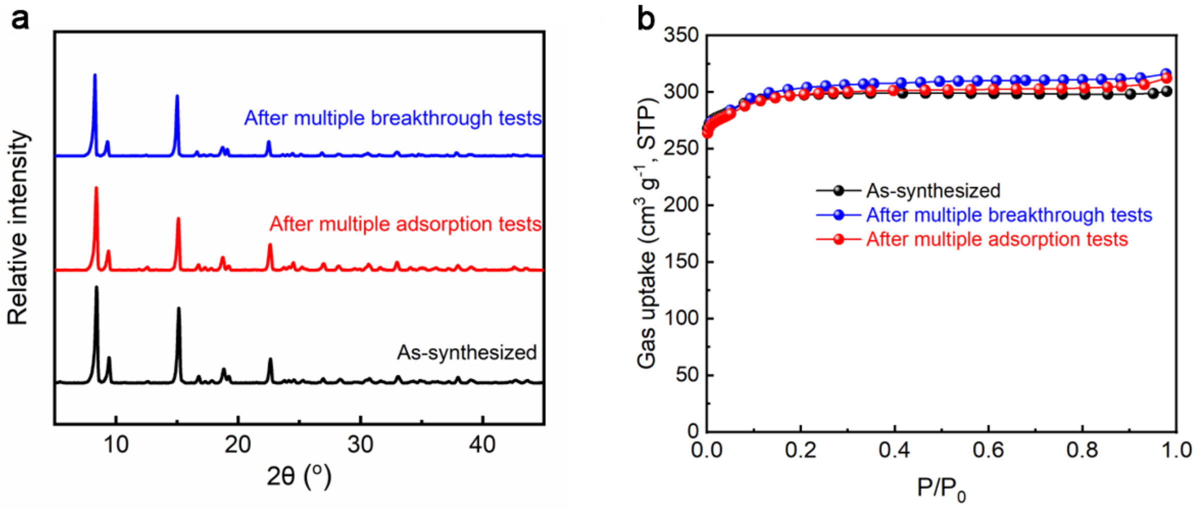

Supplementary Fig. 21 Recyclability. **a** PXRD patterns of Al-PyDC samples after multiple adsorption and breakthrough tests. **b** N_2 adsorption isotherms at 77 K of Al-PyDC after multiple adsorption and breakthrough tests.

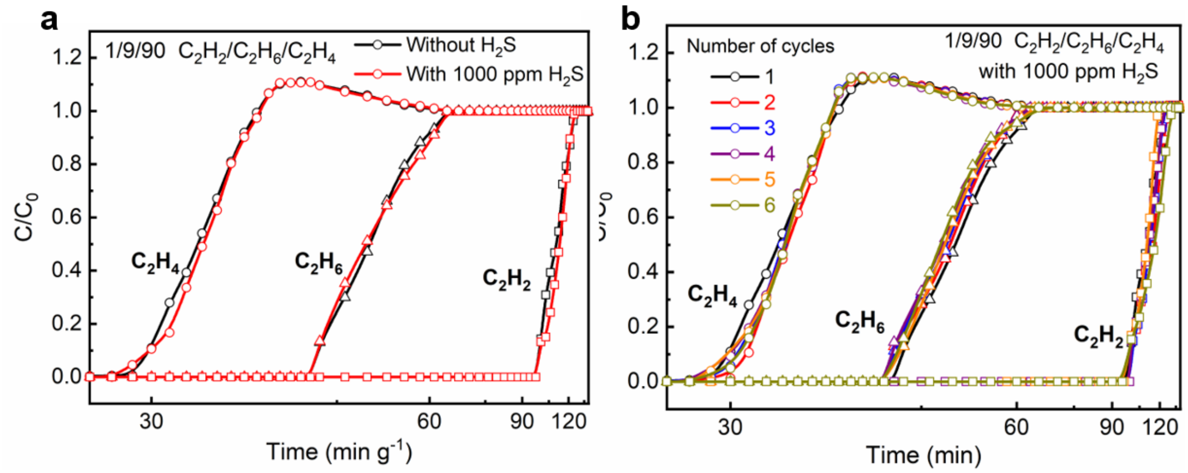

Supplementary Fig. 22 Stability and recyclability. **a** Experimental breakthrough curves of Al-PyDC for 1/9/90 $C_2H_2/C_2H_6/C_2H_4$ mixture with 1000 ppm H_2S. **b** The cycling tests of Al-PyDC for 1/9/90 $C_2H_2/C_2H_6/C_2H_4$ mixture with 1000 ppm H_2S.

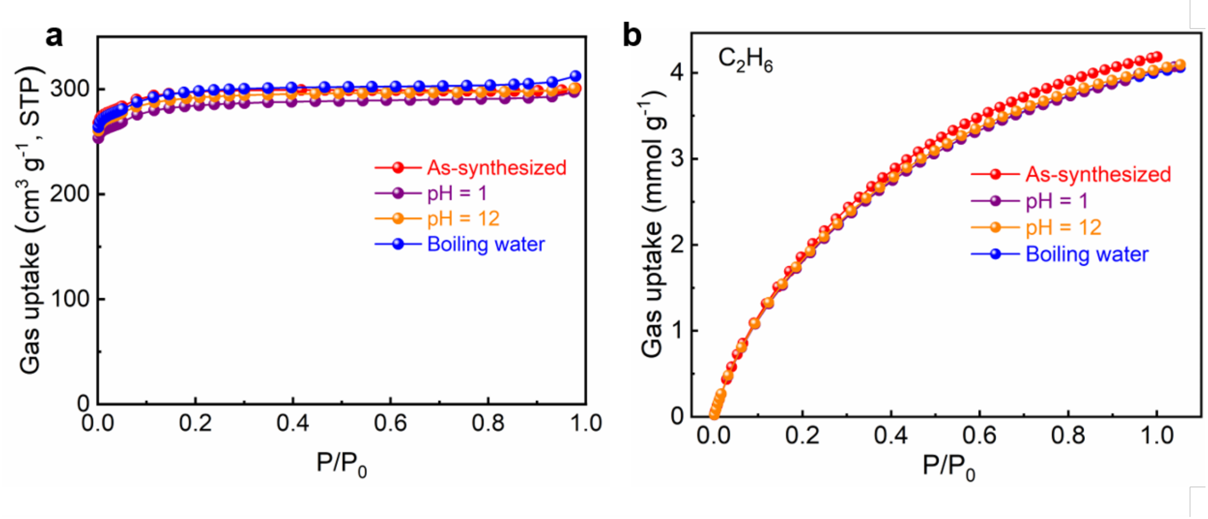

Supplementary Fig. 23 Chemical stability. a N_2 and **b** C_2H_6 adsorption isotherms of Al-PyDC samples after treatment with different conditions at 77 K and 296 K, respectively.

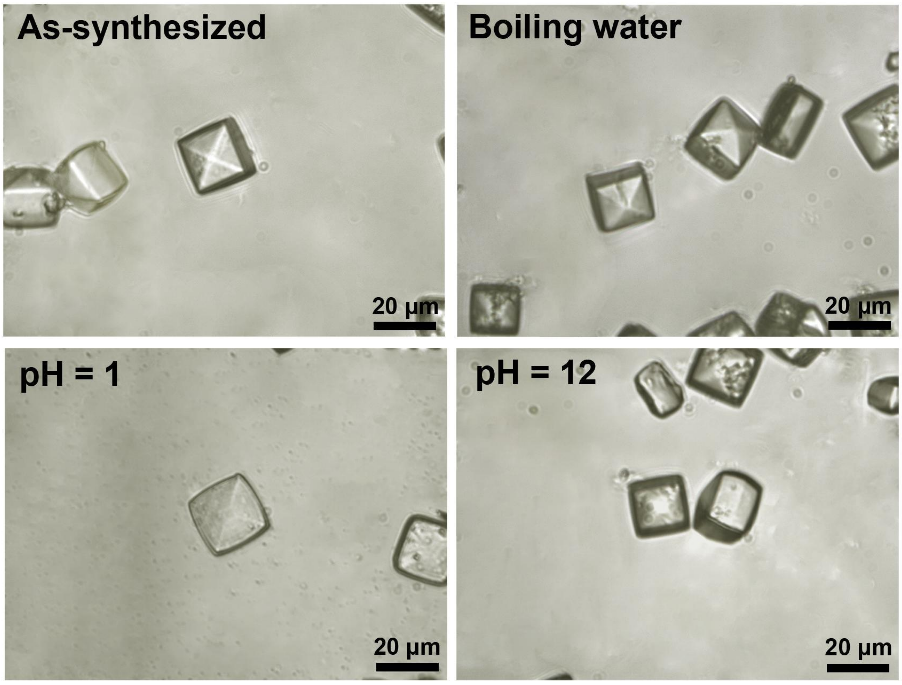

Supplementary Fig. 24 Optical microscope images. Optical microscope images of Al-PyDC crystal after treatment under different conditions.

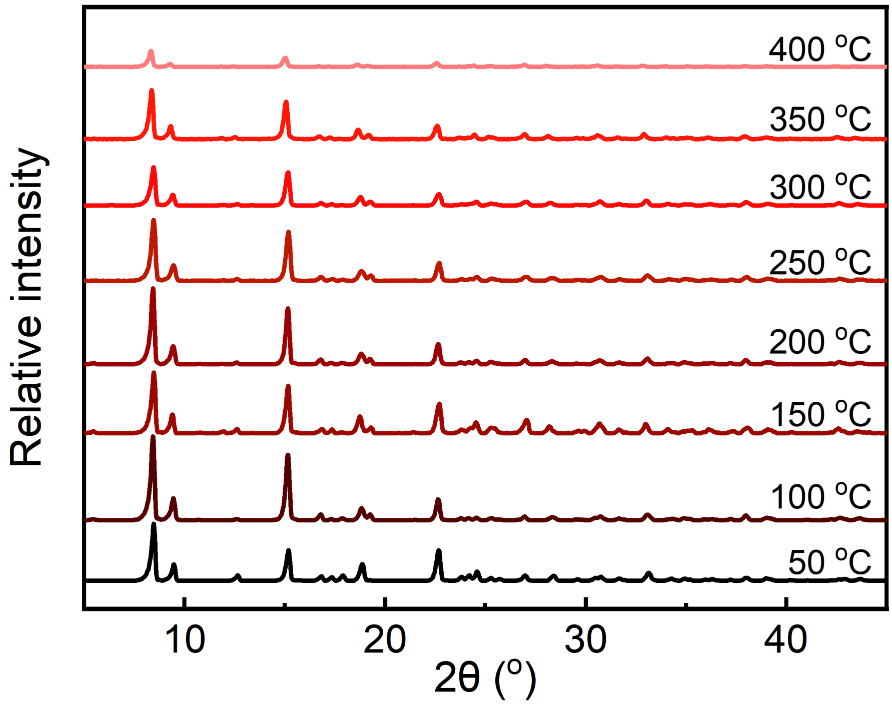

Supplementary Fig. 25 Thermal stability. Variable-temperature PXRD patterns for Al-PyDC.

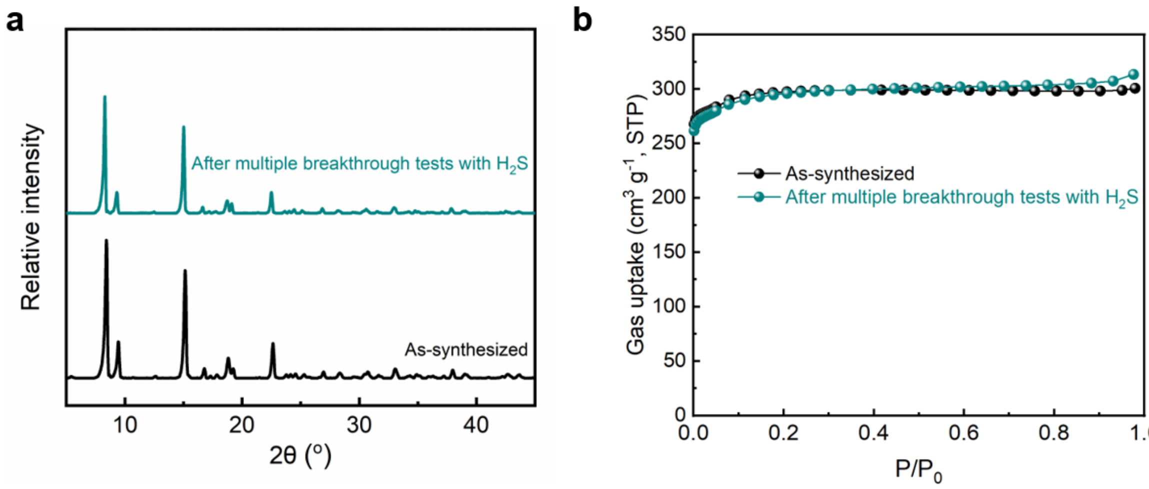

Supplementary Fig. 26 Stability. a PXRD patterns of Al-PyDC samples after multiple breakthrough tests for 1/9/90 $C_2H_2/C_2H_6/C_2H_4$ mixture with 1000 ppm H_2S. **b** N_2 adsorption isotherms at 77 K of Al-PyDC after multiple breakthrough tests for 1/9/90 $C_2H_2/C_2H_6/C_2H_4$ mixture with 1000 ppm H_2S.

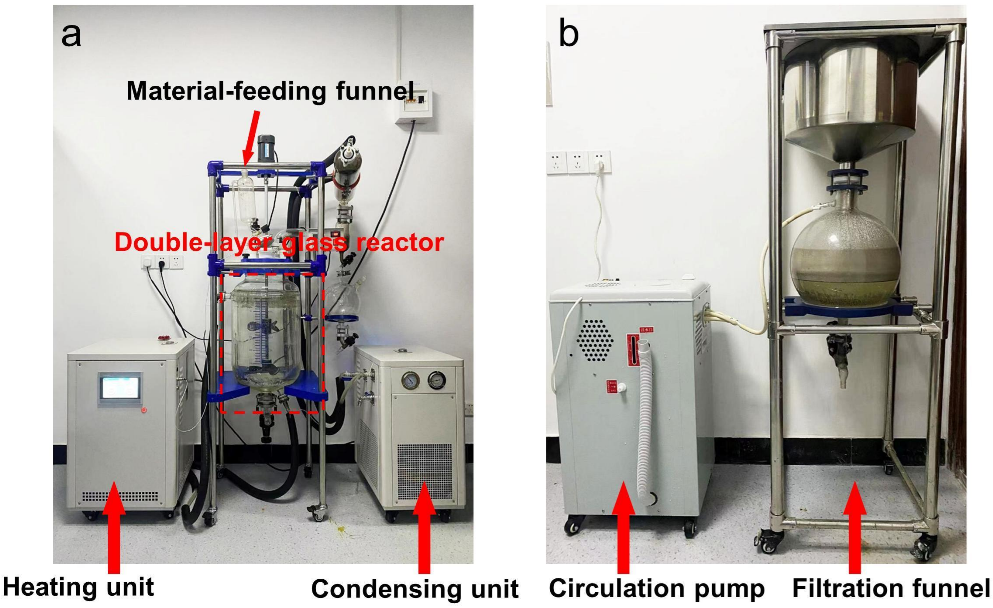

Supplementary Fig. 27 Synthesis unit. a The large scale synthesis vessel consists of a heating unit, a condensing unit, a material-feeding funnel and a 30 L double glazed reactor, where the temperature of the reactor is controlled by heating the silicone oil between the glass interlayers. **b** The 20L filtration funnel with circulation pump.

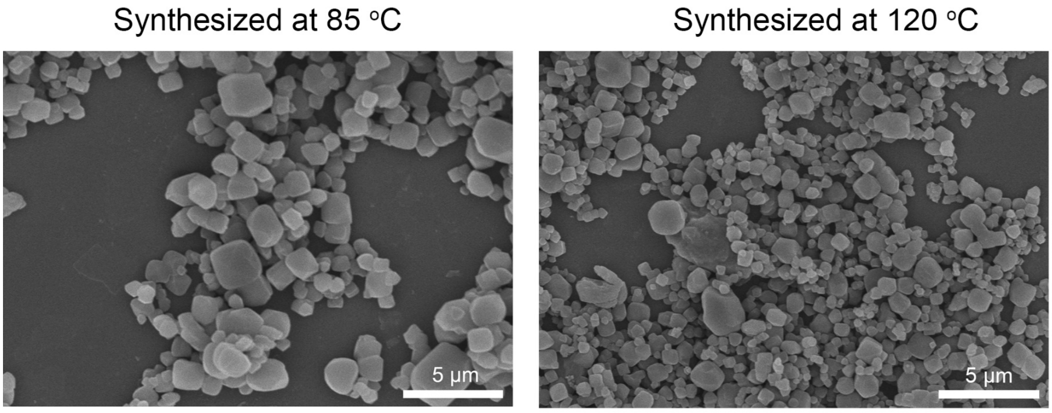

Supplementary Fig. 28 SEM images. (left) The SEM images of large-scale sample synthesized at low temperature (85 °C), and (right) the powder sample synthesized at 120 °C according to the literature[18].

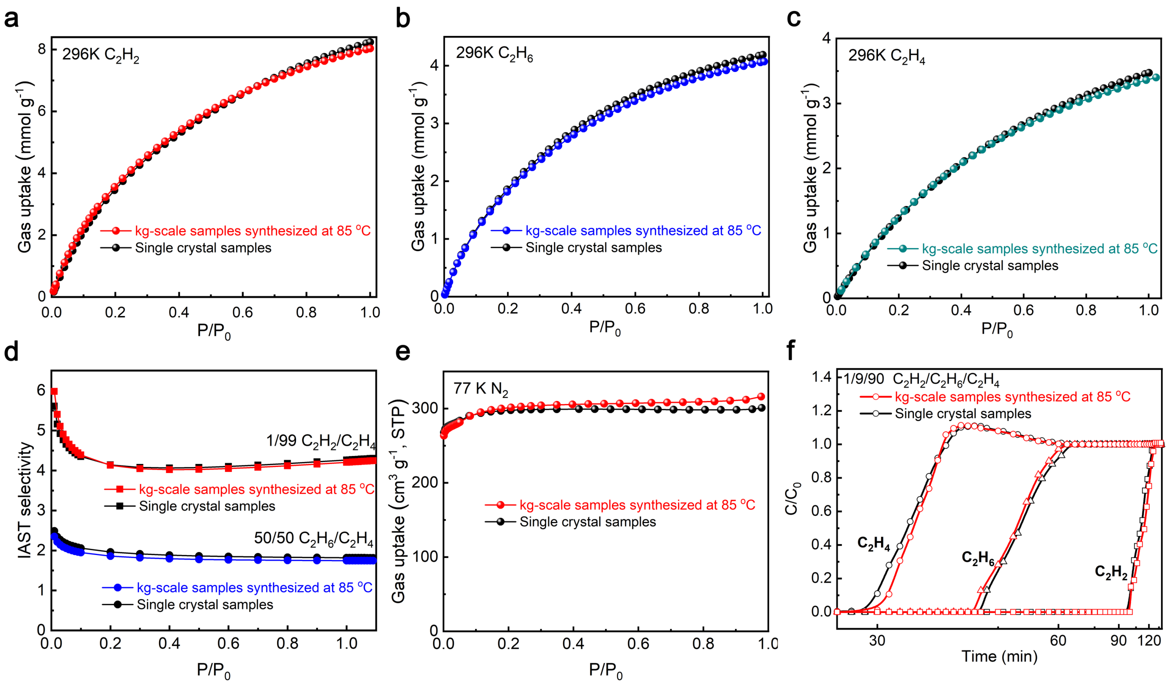

Supplementary Fig. 29 Scalability. Comparison of **a-c** C_2 gas adsorption at 296 K, **d** N_2 adsorption at 77 K, **e** IAST selectivity and **f** breakthrough experiment curves of kg-scale Al-PyDC sample synthesized at low temperature of 85 °C with those obtained from single crystal sample.

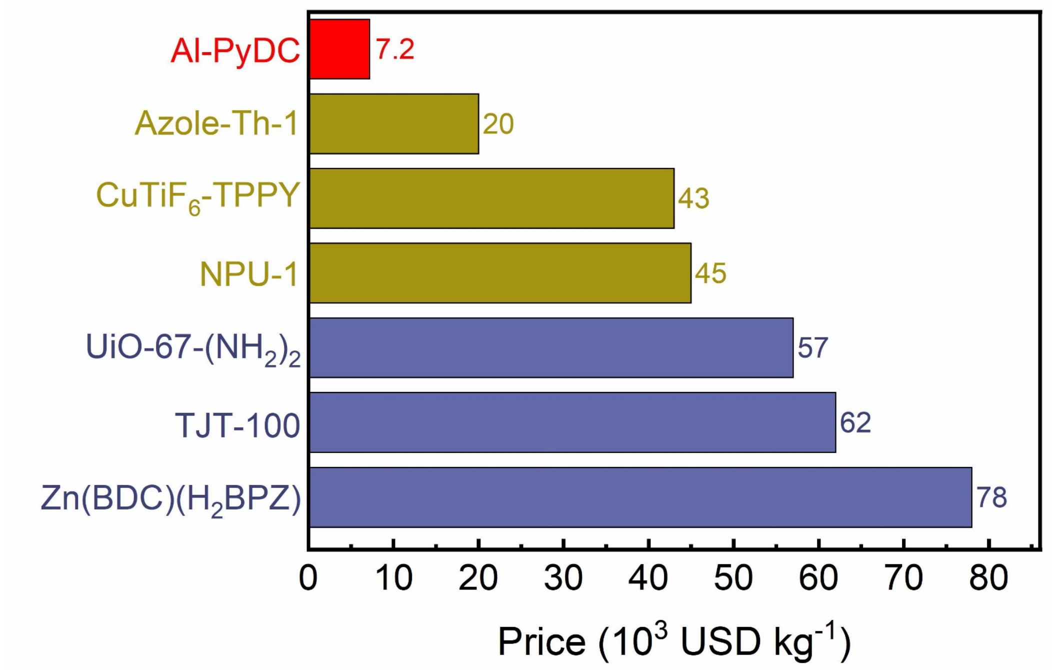

Supplementary Fig. 30 Economic feasibility. Comparison of the price of organic ligands for best-performing MOF materials. The prices were inquired by Bidepharm (China) or other companies. Note that: the values of ligand price are used only for the qualitative comparison purpose.

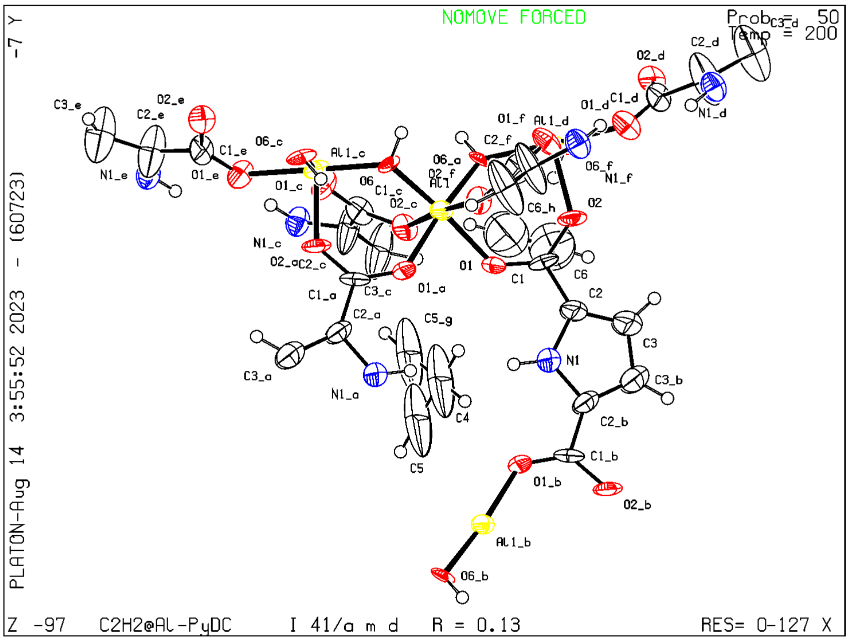

Supplementary Fig. 31 ORTEP. ORTEP drawing of the C_2H_2@Al-PyDC, produced by the checkCIF report of the International Union of Crystallography. CCDC deposit number 2242152.

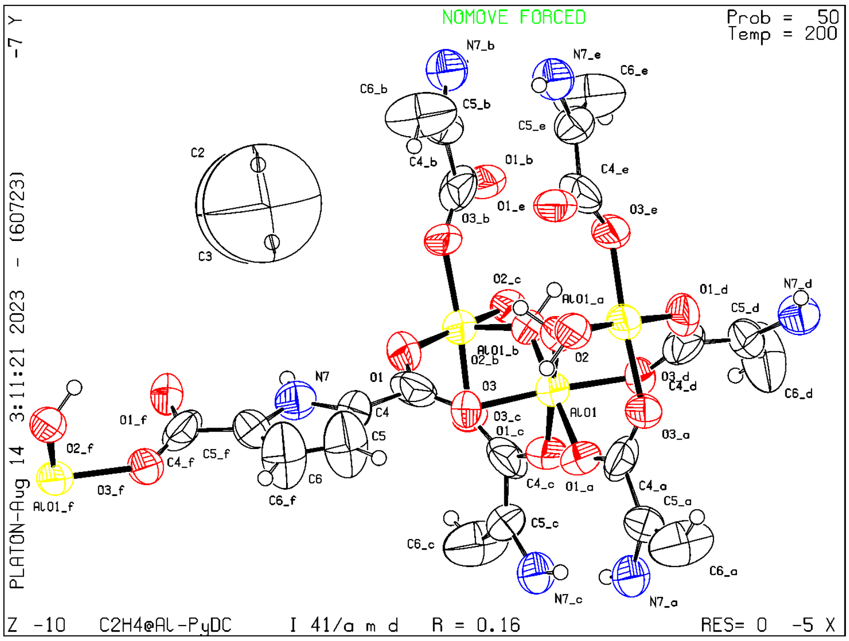

Supplementary Fig. 32 ORTEP. ORTEP drawing of the C_2H_4@Al-PyDC, produced by the checkCIF report of the International Union of Crystallography. CCDC deposit number 2242153.

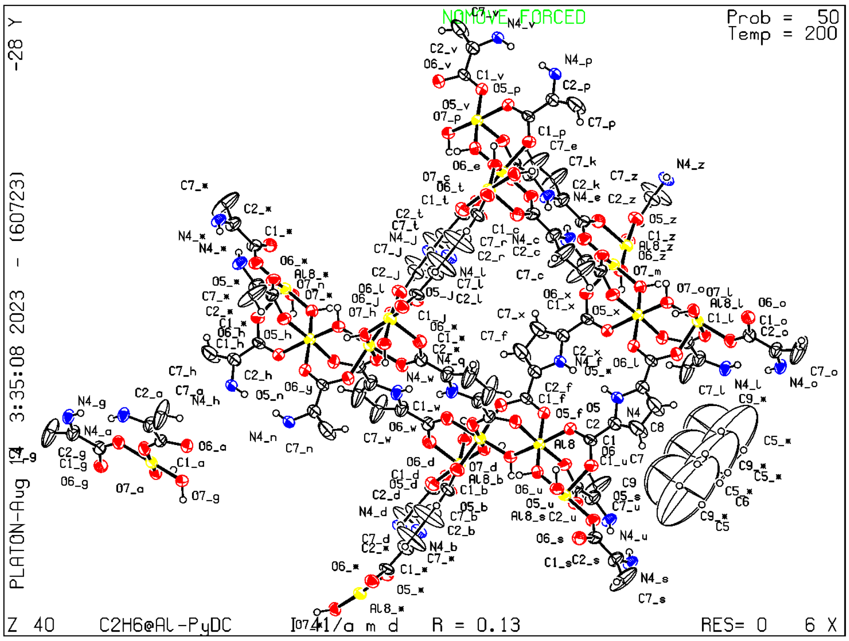

Supplementary Fig. 33 ORTEP. ORTEP drawing of the C_2H_6@Al-PyDC, produced by the checkCIF report of the International Union of Crystallography. CCDC deposit number 2242154.

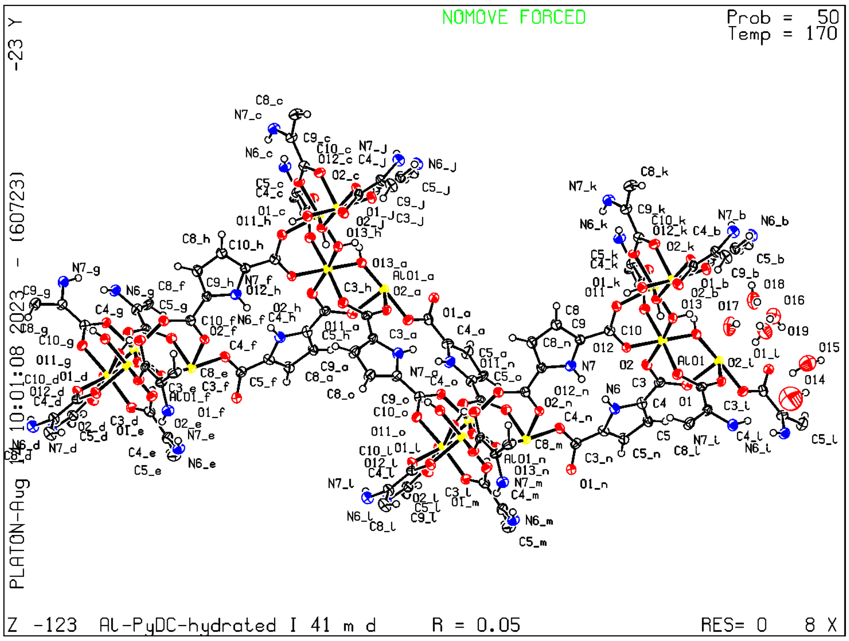

Supplementary Fig. 34 ORTEP. ORTEP drawing of the Al-PyDC-hydrated, produced by the checkCIF report of the International Union of Crystallography. CCDC deposit number 2242155.

Disclaimer: Certain commercial suppliers are identified in this paper to foster understanding. Such identification does not imply recommendation or endorsement by the National Institute of Standards and Technology, nor does it imply that the materials or equipment identified are necessarily the best available for the purpose.

Supplementary References

1. Zhang, X. et al. Selective ethane/ethylene separation in a robust microporous hydrogen-bonded organic framework, *J. Am. Chem. Soc.* **142**, 633−640 (2020).

2. Ding, Q. et al. One-step ethylene purification from ternary mixtures in a metal−organic framework with customized pore chemistry and shape. *Angew. Chem. Int. Ed.* **61**, e202208134 (2022).

3. Zhang, P. et al. Synergistic binding sites in a hybrid ultramicroporous material for one-step ethylene purification from ternary C_2 hydrocarbon mixtures. *Sci. Adv.* **8**, eabn9231 (2022).

4. Wang, G.-D. et al. One-step C_2H_4 purification from ternary $C_2H_6/C_2H_4/C_2H_2$ mixtures by a robust metal-organic framework with customized pore environment. *Angew. Chem. Int. Ed.* **61**, e202205427 (2022).

5. Gu, X.-W. et al. Immobilization of Lewis basic sites into a stable ethane-selective MOF enabling one-step separation of ethylene from a ternary mixture. *J. Am. Chem. Soc.* **144**, 2614−2623 (2022).

6. Zhu, B. et al. Pore engineering for one-step ethylene purification from a three-component hydrocarbon mixture. *J. Am. Chem. Soc.* **143**, 1485−1492 (2021)

7. Hao, H.-G. et al. Simultaneous trapping of C_2H_2 and C_2H_6 from a ternary mixture of $C_2H_2/C_2H_4/C_2H_6$ in a robust metal−organic framework for the purification of C_2H_4. *Angew. Chem. Int. Ed.* **57**, 16067−160711 (2018).

8. Xu, Z. et al. A robust Th-azole framework for highly efficient purification of C_2H_4 from a $C_2H_4/C_2H_2/C_2H_6$ mixture. *Nat. Commun.* **11**, 3163 (2020).

9. Yang, S.-Q. et al. Efficient purification of ethylene from C_2 hydrocarbons with an C_2H_6/C_2H_2-selective metal−organic framework. *ACS Appl. Mater. Interfaces* **13**, 962−969 (2021).

10. Wang, Y. et al. One-step ethylene purification from an acetylene/ethylene/ethane ternary mixture by cyclopentadiene cobalt-functionalized metal−organic frameworks. *Angew. Chem. Int. Ed.* **60**, 11350−11358 (2021).

11. Sikma, R. E. et al. Low-valent metal ions as MOF pillars: a new route toward stable and multifunctional MOFs. *J. Am. Chem. Soc.* **143**, 13710−13720 (2021).

12. Qian, X. et al. Structure stability of metal-organic framework MIL-53 (Al) in aqueous solutions. *Int. J. Hydrogen Energy.* **38**, 16710−16715 (2013).

13. Huang, Y., Qin, W., Li, Z. & Li, Y. Enhanced stability and CO_2 affinity of a UiO-66 type metal−organic framework decorated with dimethyl groups. *Dalton Trans.* **41**, 9283−9285 (2012).

14. Park, K. S. et al. Exceptional chemical and thermal stability of zeolitic imidazolate frameworks. *Proc. Natl. Acad. Sci.* **103**, 10186−10191 (2006).
15. Wang, K., Li, Y., Xie, L.-H., Li, X. & Li, J.-R. Construction and application of base-stable MOFs: a critical review. *Chem. Soc. Rev.* **51**, 6417−6441 (2022).
16. Feng, D. et al. Kinetically tuned dimensional augmentation as a versatile synthetic route towards robust metal−organic frameworks. *Nat. Commun.* **5**, 5723 (2014).
17. Wang, B. et al. Highly stable Zr(IV)-based metal−organic frameworks for the detection and removal of antibiotics and organic explosives in water. *J. Am. Chem. Soc.* **138**, 6204−6216 (2016).
18. Cho, K. H. et al. Rational design of a robust aluminum metal-organic framework for multi-purpose water-sorption-driven heat allocations. *Nat. Commun.* **11**, 5112 (2020).
